# An EMT-related genes signature as a prognostic biomarker for patients with endometrial cancer

**DOI:** 10.1186/s12885-023-11358-4

**Published:** 2023-09-18

**Authors:** Yonghui Yu, Yiwen Zhang, Zhi Li, Yongshun Dong, Hongmei Huang, Binyao Yang, Eryong Zhao, Yongxiu Chen, Lei Yang, Jiachun Lu, Fuman Qiu

**Affiliations:** 1 State Key Lab of Respiratory Disease, Institute for Chemical Carcinogenesis, Collaborative Innovation Center for Environmental Toxicity, School of Public Health, Guangzhou Medical University, 1 Xinzao Road, Xinzao, Panyu District, Guangzhou, 511436 China; 2 Department of Obstetrics and Gynecology, Key Laboratory for Major Obstetric Diseases of Guangdong Province, The Third Affiliated Hospital of Guangzhou Medical University, Guangzhou, Guangdong China; 3 Innovation Center for Advanced Interdisciplinary Medicine, The Fifth Affiliated Hospital of Guangzhou Medical University, Guangzhou, Guangdong China; 4 Department of Obstetrics and Gynecology, Guangzhou Women and Children’s Medical Center, Guangzhou, Guangdong China; 5 Department of Gynaecology and Obstetrics, Guangdong Women’s and Children’s Hospital, Guangzhou, Guangdong China

**Keywords:** Endometrial cancer, EMT, Biomarker, Prognosis

## Abstract

**Background:**

The epithelial-mesenchymal transition (EMT) plays an indispensable role in the development and progression of Endometrial cancer (EC). Nevertheless, little evidence is reported to uncover the functionality and application of EMT-related molecules in the prognosis of EC. This study aims to develop novel molecular markers for prognosis prediction in patients with EC.

**Methods:**

RNA sequencing profiles of EC patients obtained from The Cancer Genome Atlas (TCGA) database were used to screen differential expression genes (DEGs) between tumors and normal tissues. The Cox regression model with the LASSO method was utilized to identify survival-related DEGs and to establish a prognostic signature whose performance was evaluated by Kaplan–Meier curve, receiver operating characteristic (ROC) and calibration curve. Eventually, functional enrichment analysis and cellular experiments were performed to reveal the roles of prognosis-related genes in EC progression.

**Results:**

A total of 540 EMT-related DEGs in EC were screened, and subsequently a four-gene risk signature comprising *SIRT2*, *SIX1*, *CDKN2A* and *PGR* was established to predict overall survival of EC. This risk signature could serve as a meaningfully independent indicator for EC prognosis via multivariate Cox regression (HR = 2.002, 95%CI = 1.433–2.798; *P* < 0.001). The nomogram integrating the risk signature and clinical characteristics exhibited robust validity and performance at predicting EC overall survival indicated by ROC and calibration curve. Functional enrichment analysis revealed that the EMT-related genes risk signature was associated with extracellular matrix organization, mesenchymal development and cellular component morphogenesis, suggesting its possible relevance to epithelial-mesenchymal transition and cancer progression. Functionally, we demonstrated that the silencing of *SIX1*, *SIRT2* and *CDKN2A* expression could accelerate the migratory and invasive capacities of tumor cells, whereas the downregulation of *PGR* dramatically inhibited cancer cells migration and invasion.

**Conclusions:**

Altogether, a novel four-EMT-related genes signature was a potential biomarker for EC prognosis. These findings might help to ameliorate the individualized prognostication and therapeutic treatment of EC patients.

**Supplementary Information:**

The online version contains supplementary material available at 10.1186/s12885-023-11358-4.

## Introduction

Endometrial cancer (EC), one of the most common gynecological malignancies, has an increasing incidence and disease-associated mortality worldwide and in China over the past decade [1, 2]. The histological grading of endometrial cancer is mainly based on the range of firmness in the tumor, and the grading criteria are as follows: Grade 1, solid growth pattern ≤ 5%; Grade 2, solid growth pattern 6% ~ 50%; Grade 3, solid growth pattern > 50%. In addition, depending on the area of the nucleus, the final tumor grade can be increased by one [3]. According to the pathogenesis and histological characteristics, endometrial cancer can be commonly divided into estrogen-dependent (type I) and estrogen-independent (type II) [4]. The type I EC is relevant to unopposed estrogen stimulation, comprising low-grade cells that are more common and have a favorable prognosis, whereas the type II EC is not estrogen driven, comprising high-grade cells that are less common and have an unfavorable outcome. Most of the type I EC were endometrioid adenocarcinoma, and a few were mucinous adenocarcinoma, and the type II EC include serous carcinoma, clear cell carcinoma and carcinosarcoma [4].

EC is commonly diagnosed at an early stage as it presents with symptoms in its initial phases [5]. 67.5% of women with EC were diagnosed with localized disease with 5-year survival rate of 94.9%. However, a large proportion of EC patients diagnosed with metastatic state, the 5-year survival rate plummets to 18.4%, which disproportionately adds to the overall mortality rate [6]. Despite being an increasingly prevalent malignancy, progress towards improving the survival rate of women affected by EC has been limited over the years [7]. Therefore, discovering molecular biomarkers linked to EC metastasis and prognosis becomes a critical need for effective disease management.

The transformation of epithelial cells to mesenchymal cells has convincingly been known to enhance cell invasiveness, making epithelial-mesenchymal transition (EMT) a key process in the development and progression of malignant tumors [8]. EMT is the dynamic process of transforming malignant epithelial cells into mesenchymal cells. During this process, mesenchymal markers such as N-cadherin [9] and vimentin [10] are upregulated while epithelial markers like E-cadherin [9]) are downregulated. EMT is found to be highly associated with poorer prognosis in patient groups with multiple cancers, including breast [11], lung [12], head and neck [13], or ovarian cancer [14]. EMT-related signaling pathways have thus become an attractive therapeutic target [15], particularly inextricably linked to the progression of EC [16]. In fact, recent studies have found that EMT-related molecular markers are significantly relevant to poor clinical outcomes in patients with EC [17]. As a result, EMT-related genes may be capable of serving as predictors of clinical prognosis for patients with EC.

In the current study, through mining EC High throughput sequencing data from The Cancer Genome Atlas (TCGA) database, we aim to construct a risk signature based on EMT-related genes to predict the survival of EC patients and to analyze the biological function of EMT-related genes.

## Materials and methods

### EC Datasets and EMT-Related Genes

By searching for the keywords “Epithelial-to-mesenchymal transition” or “EMT” in the GeneCards (https://www.genecards.org/; access to 2022–4-28) which is comprehensive, authoritative compendium for searchable human gene annotations, genes associated with EMT were screened by Category with “Protein Coding” and relevance score > 5.0 for inclusion and there were 701 candidate genes in the follow-up study. Gene expression profiles and clinical information of EC patients of TCGA database were downloaded from the UCSC Xena public platform (http://xena.ucsc.edu/). The RNA-seq data was normalized by log2(x + 1). There were including 543 EC malignant tumor samples and 35 normal samples for comparison of gene expression differences. After excluding cases without complete survival time and status information, 542 EC patients were included for subsequent survival analysis (shown in Table S1). Six datasets including GSE56087, GSE106191, GSE17025, GSE115810, GSE36389 and GSE63678 were collected from the GEO database (http://www.ncbi.nlm.nih.gov/geo/) for comparisons of gene expression between cancer and normal tissues. The basic information of six GEO datasets was showed in Table S2.

### Establishment of genes risk signature

Differentially expressed genes (DEGs) between cancer and normal tissues were generated using nonparametric Wilcoxon rank-sum test and corrected with the Benjamini–Hochberg procedure. Genes with an adjusted *P* < 0.05 were considered as differentially expressed genes. Univariate Cox regression analysis was applied to identify candidate prognostic genes. LASSO-Cox regression analysis was performed to filter and select genes associated with survival based on the best value of lambda. Subsequently, the stepwise multivariate Cox regression analysis was used to further select candidate genes. A risk signature was established according to the stepwise Cox regression coefficient multiplied by its gene expression. The risk score formula was constructed as follows: risk score = $\sum\limits_{{{\text{i}} = 1}}^{N} {(Exp_{i} * Coe_{i} )}$(N: the number of selected EMT-related genes; $\mathrm E{\text{xp}}_{\mathrm i}$:the expression value of each EMT-related gene;$\mathrm C{\text{oe}}_{\mathrm i}$:multivariate Cox regression coefficient).

Patients with EC were assigned to the high-risk group (*N* = 271) and the low-risk group (*N* = 271) according to the median risk scores. Kaplan–Meier curves were used to evaluate the correlation between genes expression and overall survival (OS), as well as progression-free interval (PFI) and disease-free interval (DFI), tested by Log-Rank test. If the median survival time (MST) between groups could not be calculated, the mean survival time was used instead. A nomogram was constructed to investigate the probability of 1-, 3-, 5- and 10-year OS of EC. The calibration curve was plotted to assess whether the predict probability was in agreement with actual rate in the nomogram.

### Functional enrichment analysis

The GENIE3 algorithm of R package was used to construct a gene regulatory network, which was visualized by Cytoscape 3.7.1. The top 200 hub genes were obtained with Maximal Clique Centrality (MCC) algorithm by CytoHubba, a plug-in of Cytoscape. The Gene ontology (GO) analysis was initially carried out using R clusterProfiler. Then GO and Kyoto encyclopedia of genes and genomes (KEGG) pathway enrichment analyses [18] were also validated via Metascape (http://metascape.org). GO terms and KEGG pathways with BH-corrected P < 0.05 were considered as significant.

### Tissue specimens

Forty-two paired EC and their adjacent non-cancer tissues from EC patients who provided informed written consent, were obtained from Affiliated Hospitals of Guangzhou Medical University. The study was approved by the Ethics Committee of Guangzhou Medical University. The clinicopathological characteristics for these patients were presented in Table S3.

### Cell culture

Human EC cell lines Ishikawa and HEC-1-B were purchased from Biospecies (Guangzhou, China). The Ishikawa and HEC-1-B cells were cultured using RPMI-1640 medium (No.C11875500BT, Thermo Fisher scientific, Beijing, China). The growth culture medium was supplemented with 10% fetal bovine serum (No.10099141C, Life Technologies, Auckland, New Zealand) and penicillin–streptomycin (No. 15140122, Life Technologies, Grand Island, USA) in the moist incubator at 37 °C with 5% CO_2_.

### Cell transfection

Small interfering RNAs (siRNAs) were designed, synthesized and obtained from GenePharma Co, Ltd. (No. A10001, Suzhou, China). The siRNA sequences are shown in Table S4. The introduction of plasmids was accomplished by GP-transfect-Mate (No. G04008, GenePharma, Suzhou, China) as demanded, and cells were harvested 24 h after transfection.

### Quantitative real-time PCR

Total RNA was extracted from endometrial cancer cells with the help of TRIzol reagent (No.15596018, Life Technologies, Carlsbad, USA), and then reverse transcribed into cDNA utilizing PrimeScript RT reagent Kit with gDNA Eraser kit (No. RR047A, Takara, Dalian, China). The qPCR reactions were conducted with SYBR Premix Ex Taq II (No. RR820A, Takara, Dalian, China) on StepOne Plus Real Time PCR System (Life Technologies, Carlsbad, USA). The qRT-PCR thermal profile started with an initial denaturation at 95 °C for 30 s, followed by 40 cycles at 95 °C for 5 s and 60 °C for 30 s. Applying the 2^−△△Ct^ method, relative gene expression was analyzed. *β-actin* was performed as an internal reference to normalize the expression levels of each gene. Primers are shown in Table S5.

### Transwell assay

Transwell (No.3422, Corning, Kennebunk, USA) and Matrigel invasion chambers (No.354480, Corning, Kennebunk, USA) were applied to detect cell migration and invasion. Non-serum medium containing transfected cells (1 × 10^5^/well) was added to the upper insert, whereas 20% serum-contained medium was added into the lower insert. After incubation at 37 °C for 24 h, cells remained in upper chambers were scrubbed off by the cotton swab, and migrated cells on the bottom surface were fixed with 4% PFA and stained with 0.1% crystal violet. Stained cells were captured utilizing the optical microscope and Image-Pro Express software. The area occupied by the cells in the figure was obtained using the ImageJ.

### Wound healing assay

A monolayer of cells was cultured in 96-well ImageLock plates (No.4379, Essen BioScience, Ann Arbor, USA). The wound space was created by WoundMaker (Essen BioScience, Ann Arbor, USA). After being washed with PBS, the cells were incubated with 10% serum-contained medium and permitted to migrate for 48 h. Micrographs were taken and wound space were measured every six hours using IncuCyte ZOOM Live-Cell Analysis System (Essen BioScience, Ann Arbor, USA).

### Immunohistochemistry (IHC)

In order to explore the protein expression levels of prognostic genes in EC, immunohistochemical figures of *SIRT2*, *SIX1*, *CDKN2A* and *PGR* in EC and normal endometrium tissues were obtained from The Human Protein Atlas (https://www.proteinatlas.org/). *SIX1*, *SIRT2*, *CDKN2A* and *PGR* were incubated with antibodies HPA001893, HPA011165, CAB000093 and HPA004751, respectively.

### Western blot

Western blot analysis was performed as previously described [19]. Total protein in cells or tissues was extracted by RIPA solution (GenePharma) and the protein concentration was measured by BCA Protein Assay Kit (Beyotime). Then, equal amounts of protein samples were loaded into each well and separated by 10% SDS-PAGE and then transferred onto a PVDF membrane (Millipore, Billerica, MA, USA). Next, the membrane was cultivated with primary antibodies against SIRT2 (#AF5256, 1: 1000, Affinity Biosciences, China), SIX1 (#DF4129, 1:1000, Affinity Biosciences, China), CDKN2A (#AF5484, 1:500, Affinity Biosciences, China), PGR (#AF6106, 1: 1000, Affinity Biosciences, China) and GAPDH (ab181603, 1:1 000, abcam) at 4 °C overnight. The membrane was washed with PBST for three times. Then the membrane was incubated with the horseradish peroxidase (HRP)-conjugated secondary antibodies (ab6721, 1:1000, abcam). The bands were visualized using a chemiluminescence detection kit (Beyotime).

### Statistical analysis

Continuous variables were shown as mean ± standard deviation (SD) and categorical variables were expressed as counts (percentages). To calculate the false discovery rate (FDR) for the LASSO-Cox results, the empirical extension of the lasso penalty method was execute proposed by previous evidence [20]. Before meta-analysis, the genes expression values from the TCGA and 6 different GEO datasets were normalized with “scale” function and performed batch effect corrections with “ComBat” function using “sva” R package. Meta-analysis was performed with “meta” in R software. In the heterogeneity test, if the *P* value ≥ 0.05 and I^2^ ≤ 50%, the fixed effect model will be employed; whereas if the *P* value < 0.05 and I^2^ > 50%, then a random effect model will be utilized. All statistical analyses were performed using R. software version 4.0.2 and *P* < 0.05 was considered as statistically significant.

## Results

### Four EMT-related genes were selected in EC

**Fig. 1:**
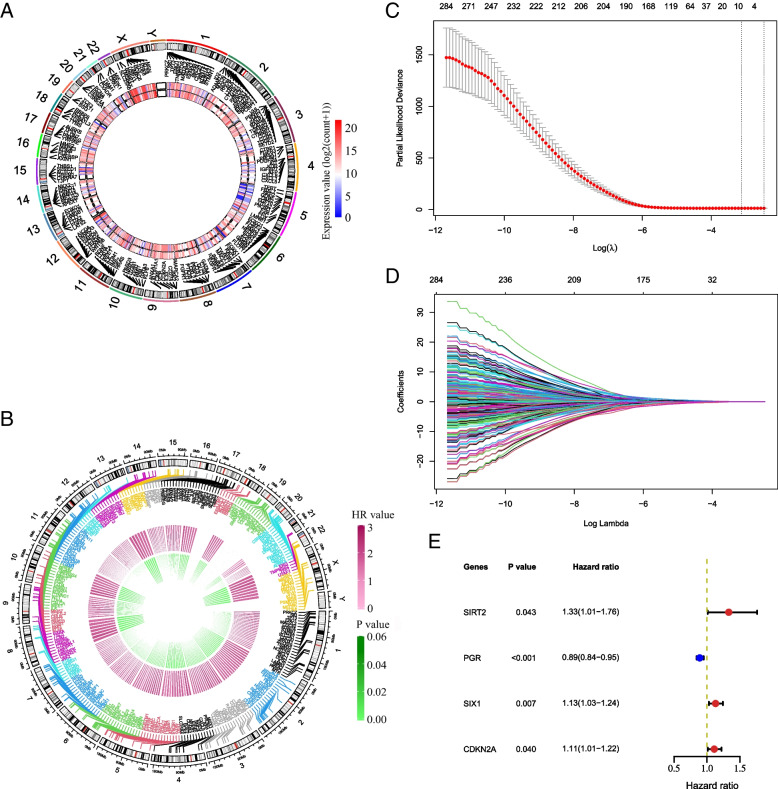
Screening EMT-related genes used for constructing the risk signature for EC. **A** The circle shows that these 540 EMT-related genes are differentially expressed between cancer and normal tissues. The inner layer presents the genes expression in cancer tissues, and the outer layer shows the genes expression in normal tissues. **B** The circle represents 278 EMT-related genes significantly associated with EC survival. **C** The most appropriate log (Lambda) value in the LASSO model. **D** The LASSO coefficient profiles of the EMT-related prognostic genes. **E** Multivariate Cox regression analysis was performed and four EMT-related genes (SIRT2, SIX1, CDKN2A and PGR) were selected to construct the risk signature. Blue square: HR < 1; red square: HR > 1; green bar: HR (95%CI)

Based on the inclusion criteria, 701 EMT-related genes were searched in the GeneCards. Among them, there were 700 candidate genes which had gene expression in the TCGA EC samples. A total of 540 DEGs with 181 up-regulated genes and 359 down-regulated EMT-related genes were screened from the TCGA database (Fig. 1 A). Next, univariate Cox regression analysis was performed. There were 278 EMT-related genes significantly associated with EC survival (Fig. 1 B). The details are shown in Table S6. Subsequently, these 278 EC prognosis-related genes were filtered by LASSO Cox regression analysis.

**Fig. 2:**
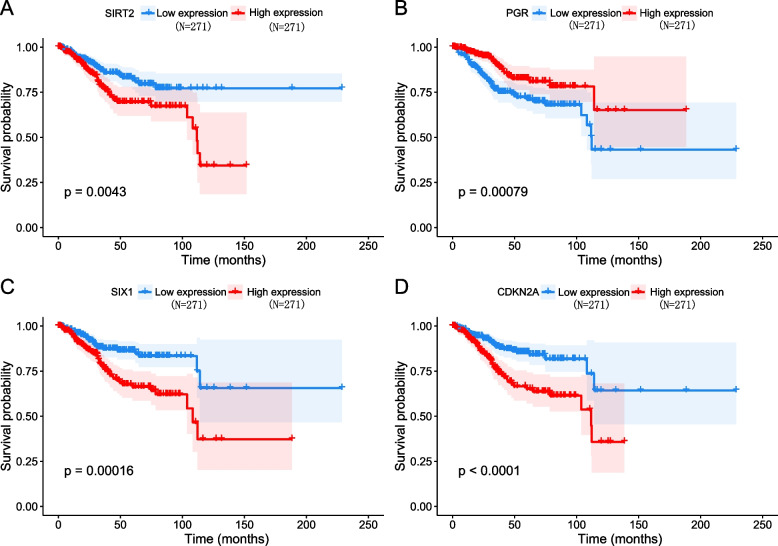
Kaplan–Meier survival curves for the four EMT-related genes in EC

The optimal parameters consisting of nine prognostic genes (i.e., *SIRT2*, *PGR*, *SPDEF*, *SIX1*, *NTS*, *ERBB2*, *CDKN2A*, *MSX1*, *ALK*) as well as their corresponding coefficients were identified (Fig. 1 C and D), which corresponded to a false discovery rate (FDR) value of less than 0.05. The results of stepwise Cox regression analysis were used to further calculate the relationship between the 9 EMT-related genes and overall survival, and finally 4 EMT-related genes (*SIRT2*, *SIX1*, *CDKN2A* and *PGR*) were identified. *SIRT2*, *SIX1* and *CDKN2A* were risk factors for poor prognosis of EC (HR > 1), meanwhile *PGR* was a protective factor (HR < 1) (Fig. 1 E). The above results were further validated according to Kaplan–Meier survival curves, and four genes were significantly associated with overall survival in EC (Fig. 2). When compared with those with low expression levels, EC patients with gene high expression had shorter mean survival time (For *SIRT2*: 33.6 months vs. 42.6 months, *P* = 0.0043; for *SIX1*: 36.1 months vs. 40.1 months, *P* = 0.00016; for *CDKN2A*: 33.3 months vs. 42.9 months, *P* < 0.0001). On the contrary, patients with high *PGR* expression had longer mean survival time than those with low expression levels (39.5 months vs. 36.7 months, *P* = 0.00079).

Furthermore, the associations between 4 EMT-related genes and progression-free interval (PFI), as well as disease-free interval (DFI), were also analyzed. As shown in Supplementary Figures S2 and S3, the expression of *SIRT2*, *SIX1* and *CDKN2*A was negatively correlated with PFI, and *PGR* was positively associated with PFI. However, no remarkable association was found for these 4 genes with DFI.

Investigating the gene expression patterns in stages of EC may provide a deeper understanding of the underlying molecular mechanisms associated with disease progression, we then further analyzed the associations of 540 DEGs with EC progression, and found that among these EMT-related DEGs, there were 11 genes (including FGF10, FAP, CD82, CDK6, NRP2, EGR1, EDNRA, FZD7, KIT, JUN and HSPA1A) significantly associated with EC stage (shown in Fig S1, all *P* < 0.05). However, for the four candidates, there were no notable relationships of *SIRT2*, *SIX1*, *CDKN2A* and *PGR* with EC stage (all *P* > 0.05).

### Four EMT-related genes expression in EC tissues

**Fig. 3:**
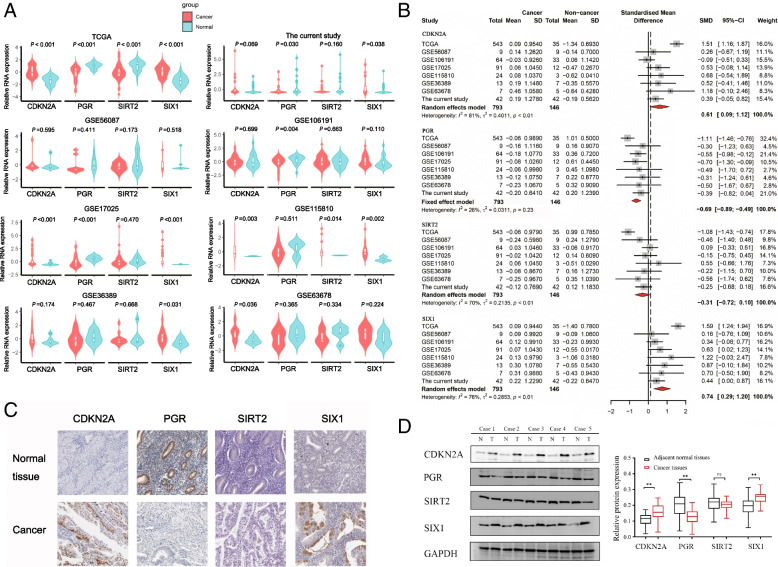
*SIRT2*, *SIX1*, *CDKN2A* and *PGR* expression in EC tissues. **A** Four EMT-related genes expression in TCGA dataset, current study and 6 GEO datasets. The gene expression was normalized by “scale” function in R. **B** Comparisons of four EMT-related genes expression between EC and normal tissues evaluated by forest-plot based on data from TCGA dataset, current study and 6 GEO datasets. Rhombus indicates the average standardized mean difference (SMD) with 95% confidence intervals. **C** Four EMT-related genes expression in EC tissue samples and corresponding non-cancer tissue samples from The Human Protein Atlas. **D** Four EMT-related genes expression in 42 paired EC tissue samples. Representative expression levels of these Four EMT-related genes in several EC patients. N, normal tissues; T, tumor tissue. ** means *P* < 0.01; ns means *P* > 0.05

Compared with normal tissues, the expression of *CDKN2A* and *SIX1* was significantly upregulated (*P* < 0.001), and oppositely the *PGR* and *SIRT2* expression was significantly downregulated in EC tissues in the TCGA dataset (*P* < 0.001, Fig. 3A). The GEO datasets and the EC tissue specimens collected from the hospital for this study served as external validation. A total of six GEO datasets were included in the meta-analysis based on the inclusion criteria. *SIX1* expression in tumor tissues was significantly higher than normal tissues in GSE17025, GSE36389, GSE11580, GSE11580 and the current study datasets. *SIRT2* expression in tumor tissues was significantly higher than normal tissues in GSE11580 datasets. *CDKN2A* expression in tumor tissues was significantly higher than normal tissues in GSE17025, GSE36389 and GSE11580 datasets. *PGR* expression in tumor tissues was significantly lower than normal tissues in GSE17025, GSE106191 and the current study datasets (Fig. 3A). Meta-analysis demonstrated that the *P*-values of *SIX1* and *CDKN2A* were less than 0.01 using a random-effects model, suggesting that their expression in EC was significantly higher than normal tissues, while the opposite was observed for SIRT2 (Fig. 3B).

To access the protein expression of the genes in the tissues, we used The Human Protein Atlas to obtain the immunohistochemistry figures for each gene. From Fig. 3C, we could distinctly notice that compared to normal endometrial tissues, *SIX1* and *CDKN2A* proteins were significantly up-regulated and *PGR* protein was significantly down-regulated in cancer tissues. However, *SIRT2* protein was not detected in both cancer and normal tissues.

To further confirm the aforementioned findings, we examined the protein levels of the four EMT-related genes in 42 pairs of EC tissues. Our results revealed a significant increase in CDKN2A and SIX1 protein expression in EC tissues compared to their corresponding paracancer tissues. Conversely, PGR protein expression was significantly decreased in the EC tissues. Unfortunately, SIRT2 expression did not show any significant variation between normal and cancerous tissues (Fig. 3D).

### Four EMT genes-based risk score model construction and assessment

**Fig. 4:**
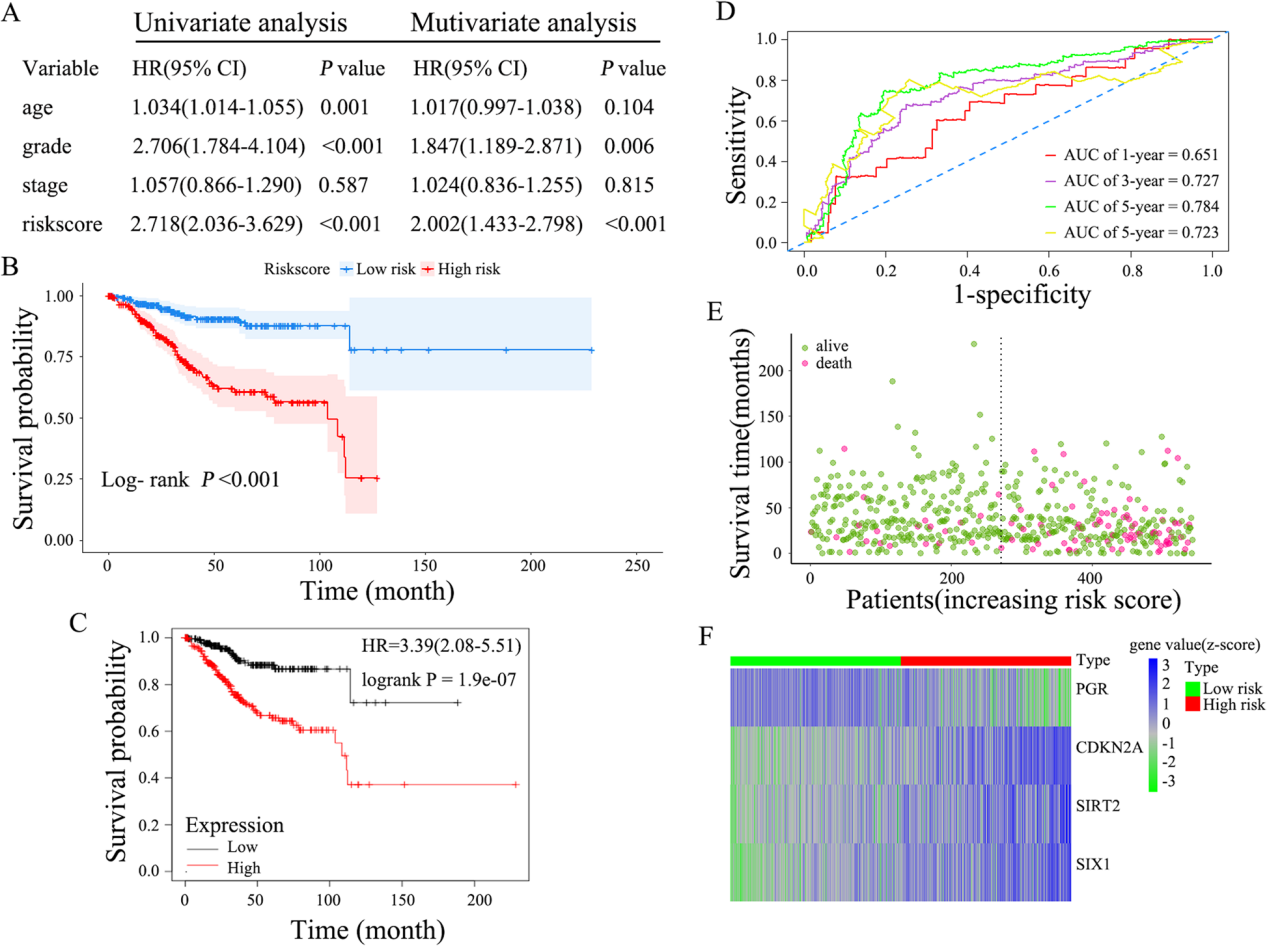
Characteristics of the four-gene risk signature and assessment. **A** Univariate and multivariate analysis of the risk signature and clinical factors. **B** Survival curves for high-risk and low-risk groups classified by the risk signature. **C** Survival analyses of risk score and EC overall survival using Kaplan–Meier Plotter. **D** ROC curves for the 1-, 3-, 5, and 10-year survival according to the four-gene risk signature. **E** Distribution of the risk score and survival status for each case. **F** The expression profiles of the four EMT-related genes between the high-risk group and low-risk group. Z-score was used to standardize the gene expression of each gene in each sample by “scale” function in R

Based on the stepwise Cox regression model, a prognostic risk score formula for EC was established. Risk score = (*SIRT2* × 0.2879) + (*PGR* × -0.1125) + (*SIX1* × 0.1228) + (*CDKN2A* × 0.1001). This formula was used to calculate the risk score for each patient. The prognostic value of the risk score was assessed by univariate and multivariate Cox regression analysis. Figure 4A demonstrates the ability of the risk score to serve as an independent prognostic indicator for EC (HR = 2.718, 95% confidence interval [CI] = 2.036–3.629. *P* < 0.001; HR = 2.002, 95% CI = 1.433–2.798, *P* < 0.001. respectively). Total 542 Patients with EC were divided into high-risk group (*N* = 271) and low-risk group (*N* = 271) based on the median risk scores. Kaplan–Meier curves showed that patients with high-risk scores had worse OS than the low-risk group (33.7 months vs. 42.5 months, *P* < 0.0001; Fig. 4B). Homoplastically, while compared to low-risk group, the patients with high-risk scores had shorter Mean survival time (for PFI: 30.1 months vs. 38.9 months, *P* < 0.0001; for DFI: 34.8 months vs. 39.7 months, *P* = 0.011; shown in Fig. S4).

To validated these findings, we further performed survival analyses using Kaplan–Meier Plotter (https://kmplot.com/analysis/index.php?p=background) with the same genes. The results also showed that the patients with high-risk score had worse OS when compared to those with low-risk score (Fig. 4C). In addition, the ROC curves were employed to appraise the predictive efficiency of this risk signature for EC. The AUCs (area under the ROC curve) of risk signature were derived as 0.651, 0.727, 0.784, and 0.723 for years 1-year, 3-year, 5-year, and 10-year, respectively (Fig. 4D). The Risk scores and survival status of each patient were depicted in Fig. S5. As shown in Fig. 4E, the number of patient deaths was found to rise (Spearman *r* = 0.271, *P* < 0.001), and the survival time was decreased with increasing risk scores (Spearman *r* = -0.167, *P* < 0.001), and the expression of *SIRT2*, *SIX1* and *CDKN2A* was increased, whereas the expression of *PGR* was decreased in high-risk group compared to those in low-risk group (Fig. 4F).

### Construction of the nomogram

**Fig. 5:**
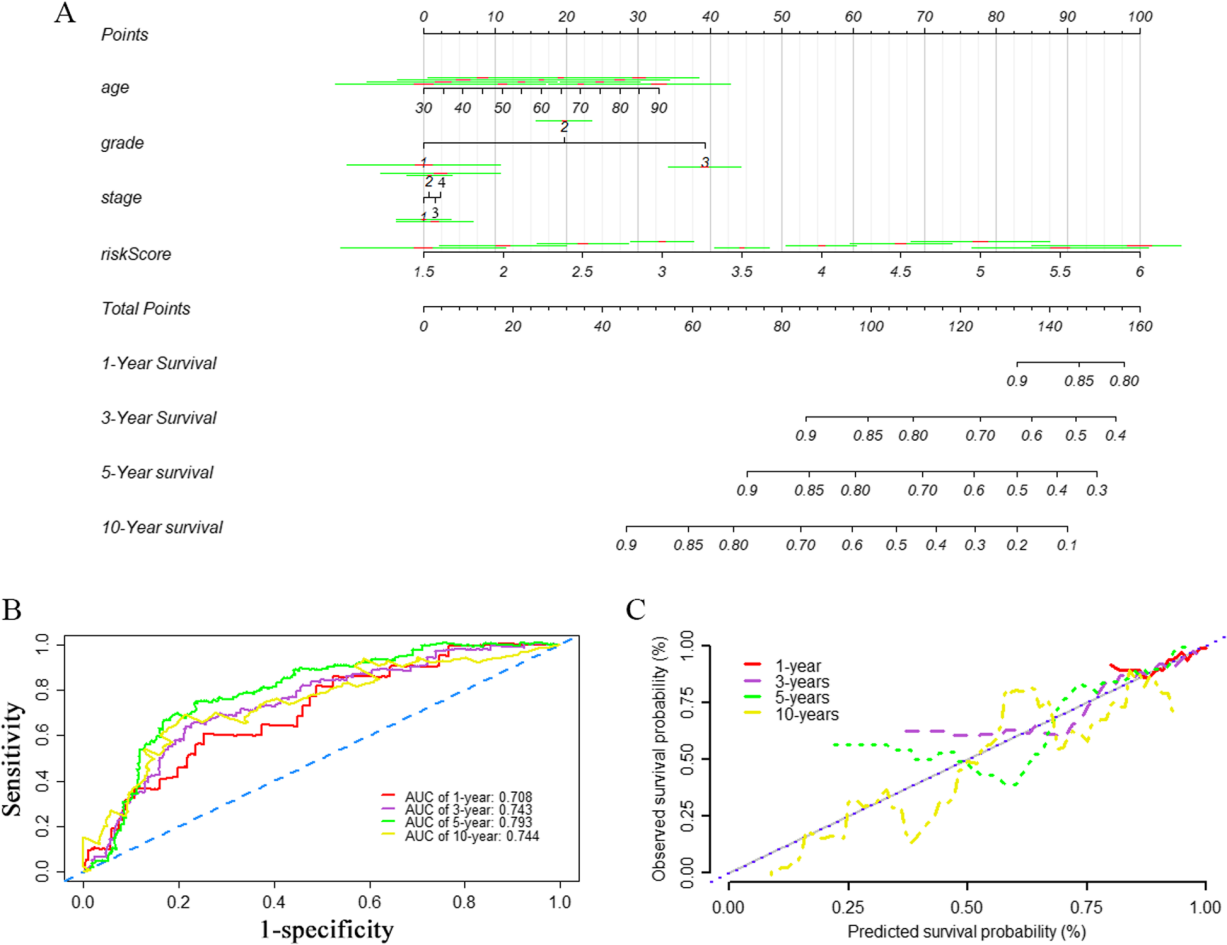
Nomogram for predicting the survival rate of EC patient and its effectiveness evaluation by using TCGA dataset. **A** A nomogram was constructed based on the risk score, age, grade, and stage for predicting survival of EC patient. The total points were calculated by drawing a vertical line from variable values to the axis labelled “Points” which can predict 1-, 3-, 5, and 10-year of overall survival (OS). The green line represents the point of each variable on the nomogram at a specified value, and the red line indicates 95%CI for the point. **B** ROC curves for evaluating the efficiency of the nomogram. **C** Calibration plot of observed and predicted survival probabilities at 1-, 3-, 5, and 10-year for the nomogram

A nomogram was constructed for visualizing survival prediction based on the multivariate Cox regression model. By integrating risk scores and clinical characteristics, including age, grade, and stage, the nomogram was implemented to predict 1-year, 3-year, 5-year, and 10-year OS of patients with EC. As shown in Fig. 5A, to utilize the nomogram, one has to locate the patient’s value for each predictor variable on the corresponding point scale, and draw a vertical line to the top point axis. The total points for all predictor variables are then located on the total point axis, and a final vertical line is drawn to the bottom outcome axis to obtain the estimated probability of the survival outcome. The AUCs for 1-year, 3-year, 5-year, and 10-year OS of the nomogram were 0.708, 0.743, 0.793, and 0.744 (Fig. 5B). The calibration curve suggested a favorable consistency between the predicted OS and the actual OS (Fig. 5C).

### Co-expression network and enrichment analysis

**Fig. 6:**
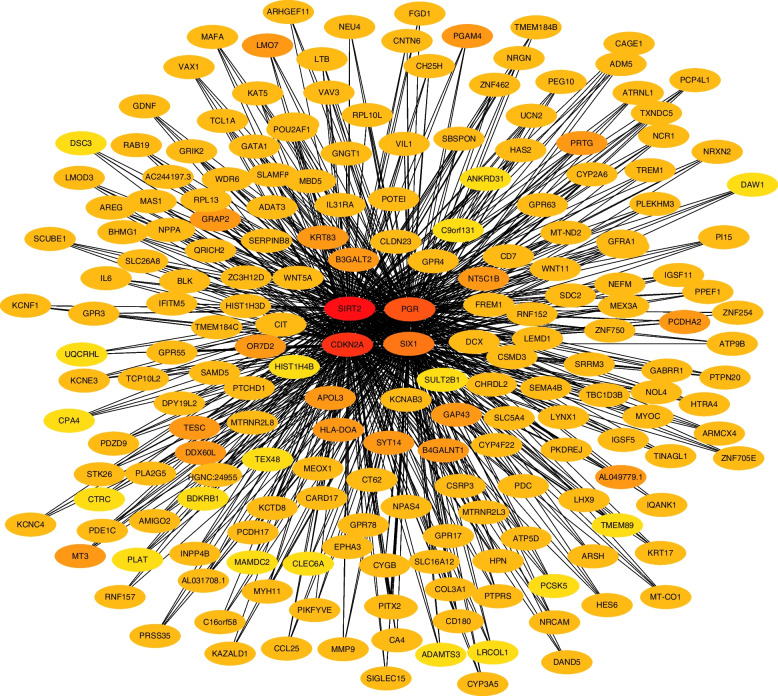
Construction of EMT-related genes regulatory network. The top 200 hub genes were obtained with MCC algorithm by CytoHubba

**Fig. 7:**
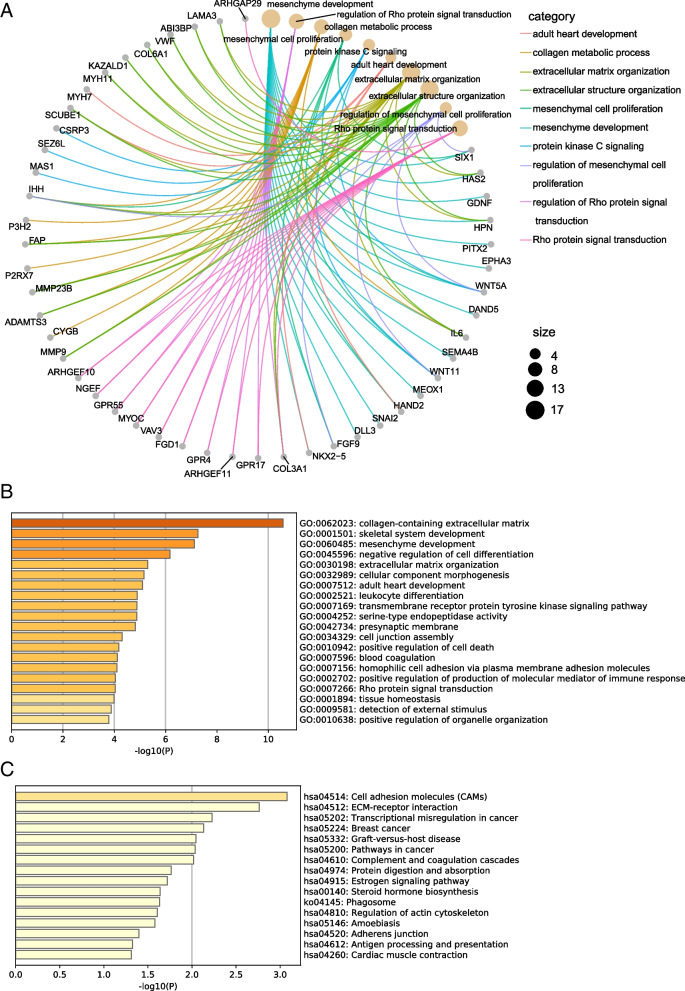
Functional enrichment analysis of the four EMT-related gene risk signature. **A** Circos plot visualize the top 10 GO enrichment analysis of co-expressed genes using clusterProfiler. **B** Bar graph for top 10 GO enrichment analysis via Metascape. **C** Bar graph for top 10 KEGG pathway analysis via Metascape

To further investigate the potential mechanisms of the four EMT-related genes in EC progression, we forecasted the gene regulatory network using GENIE3 and screened the top 200 co-expressed genes in the network via the MCC algorithm and visualized them by Cytoscape software (Fig. 6). As shown in Fig. 7A, co-expressed genes were enriched to the classification associated with extracellular matrix organization and mesenchymal development using R “clusterProfiler” package. To validate and enhance the exploration of the potential functions of the genes, we also performed GO and KEGG enrichment analysis via Metascape database. As shown in Fig. 7B and C, GO analysis revealed that genes were similarly enriched to extracellular matrix, cellular component morphogenesis related functions, while KEGG analysis indicated that genes were enriched to cell adhesion molecules, extracellular matrix receptor interaction related pathways. Additionally, we utilized STRING database (https://string-db.org/) to construct co-expression networks of the 4 EMT-related genes and conducted functional enrichment analysis using only the known interactions as input. The results revealed that the enriched pathways were still associated with EMT-related biological functions and signal pathways, such as mesenchymal cell development, mesenchymal cell differentiation, and adhesion (shown in Fig S6). Furthermore, we obtained a list of statistically significant co-expressed genes for each of the four EMT-related genes from cBioportal (https://www.cbioportal.org/) filtering with |Spearman correlation|> 0.3 and q-value < 0.05. We then constructed an alternative network containing only these co-expressed genes using Cytoscape software (Fig S7) and performed enrichment analyses using this network. We found that the alternative network is also enriched for several EMT-related pathways and processes (Fig S8).

The above results imply that four EMT-related genes are truly involved in EMT process, which can cause EC metastasis and influence cancer prognosis. Therefore, cellular experiments were performed in order to further explore the connection between the four EMT-related genes and cancer metastasis.

### *SIX1*,* SIRT2*, *CDKN2A* and* PGR* could affect cell metastasis

**Fig. 8:**
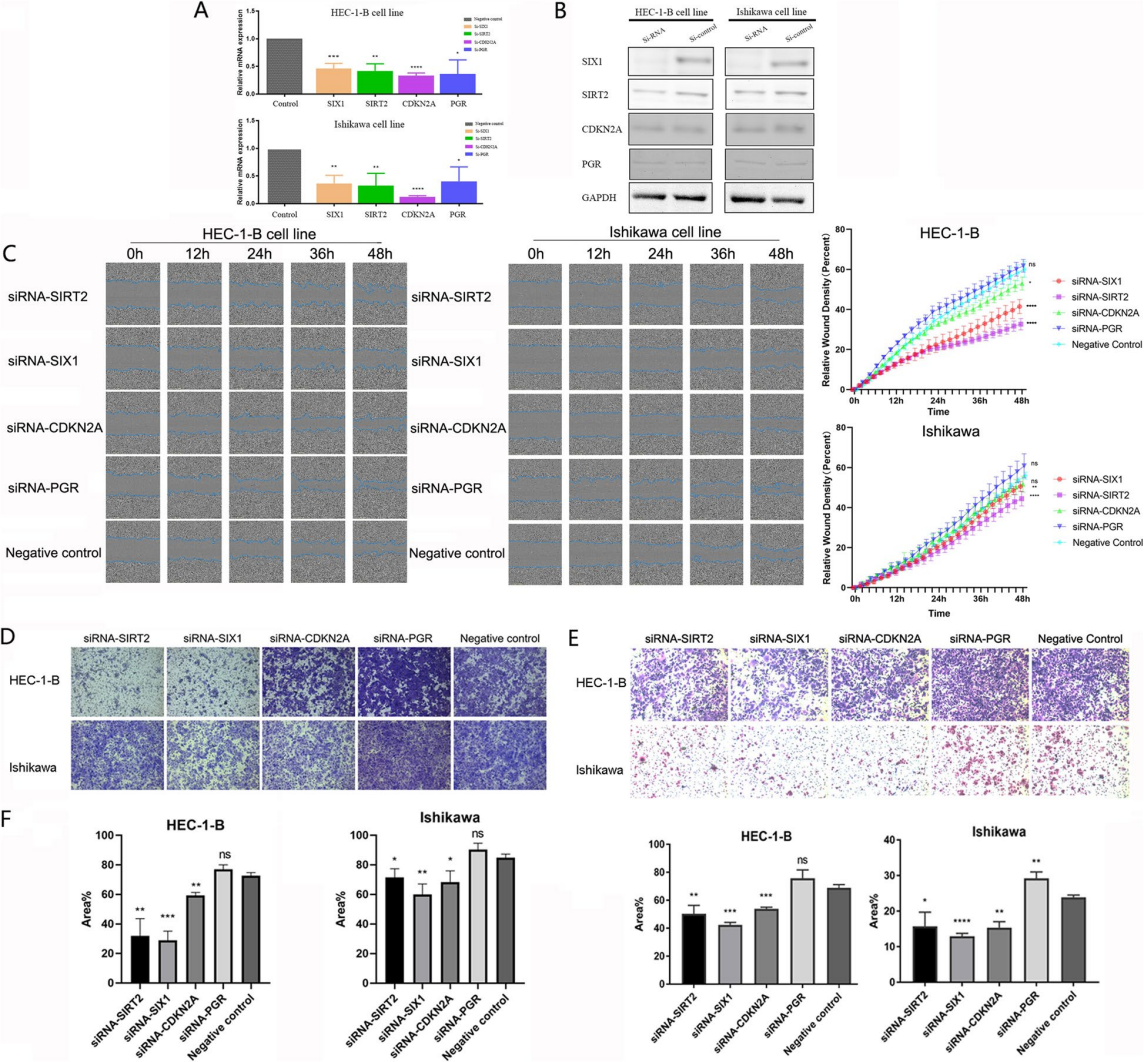
Four EMT-related genes affect EC cell metastasis. **A**-**B** Validation of silencing efficiency of four EMT-related genes in HEC-1-B and Ishikawa cell lines. **C** Migration detected by wound healing assays in HEC-1-B cell line. **D** Migration detected by wound healing assays in Ishikawa cell line. **E** Migration detected by transwell migration assay in HEC-1-B and Ishikawa cell lines. **F** Invasion detected by transwell invasion assay in HEC-1-B and Ishikawa cell lines. The means of two independent samples were compared for a statistically significant difference by the unpaired t-test. (*P < 0.05, **P < 0.01, ***P < 0.001, ns: no significant)

To verify whether four EMT-related genes (*SIX1*, *SIRT2*, *CDKN2A* and *PGR*) are associated with tumor metastasis, we performed transwell migration assays, transwell invasion assays and wound healing assays to investigate their relationship. The siRNAs of the four genes were introduced into two cell lines, HEC-1-B and Ishikawa, respectively (Fig. 8A and B). In wound healing assay, cell migration rate was significantly decreased when the genes *SIX1* and *SIRT2* were silenced, but silencing *CDKN2A* had a statistically significant negative effect on HEC-1-B cells only. In the case of *PGR*, there was no statistically significant difference in the migration rate of both cells (Fig. 8C and D). Similarly, we demonstrated that silencing of *SIX1*, *SIRT2* and *CDKN2A* substantially diminished the ability of EC cells to cross the chambers. Regrettably, there was a boost in the ability of cancer cells to cross the chambers after silencing *PGR*, but it was not significant (Fig. 8E). These results were consistent with invasion assays in HEC-1-B. Nevertheless, all four genes had a significant effect on the invasive ability of Ishikawa cells (Fig. 8F). Taken together, these results suggest a role of the four genes in migration and invasion tumoral properties of EC cells, reflecting their ability to influence cancer prognosis.

## Discussion

The prognosis for patients with early-stage EC is relatively positive. However, with metastasis being the leading cause of death [21], the prognosis for patients with advanced stages is extremely poor [22]. Therefore, it is urgent to discover biomarkers associated with metastasis and prognosis in EC. In this study, we constructed a survival prediction model for EC using gene expression data and clinical information. We identified four EMT-related genes, *SIX1*, *SIRT2*, *CDKN2A* and *PGR*, that are closely associated with EC prognosis and have a profound role in influencing the metastatic ability of EC cells. Our findings may provide a unique perspective on the progression of EC.

Advances in cancer biomarker research are impossible without technological developments. In recent years, groundbreaking advances in genomic analysis methods, such as next-generation sequencing (NGS), have greatly improved the sensitivity and high-throughput capabilities of genomic technologies [23, 24]. This has allowed researchers to extract genetic, transcriptomic, epigenomic, metabolomic, and proteomic datasets from patient cohorts. These datasets can be used as risk or prognostic factors to identify potential cancer biomarkers. Comparative statistical analysis of large multi-omics datasets can identify risk factors, allowing early detection of disease and timely intervention for better outcomes [25, 26]. In past studies, important insights into the molecular landscape of EC have been revealed. These insights were driven by the identification of biomarkers that predict disease prognosis and drug targets. Several studies based on TCGA data have found multiple genes associated with EC tumor prognosis and treatment. For example, POLE mutant tumors had significantly better progression-free survival, while tumors with high copy number had the worst prognosis [27]. *PI3K* inhibition has been successful in advanced EC, and although some limitations remain, alterations in the *PI3K /mTOR* pathway can be observed in patients [28]. A large number of molecular biomarkers of EC have been shown to correlate with clinical outcomes and to be reproducible [27, 29]. This reveals that molecular biomarker research in EC has important prospects for development. It has far-reaching implications for clinical patients, as it can be used to tailor treatment approaches to improve prognosis and achieve precision medicine. Therefore, it is expected that molecular biomarkers will be discovered that are closely related to the prognosis of EC patients.

In this study, we comprehensively analyzed the gene expression profiles and corresponding clinical information of EC patients in the TCGA database. We screened four independent EMT-related prognostic factors for EC: *SIX1, SIRT2, CDKN2A* and *PGR*. These four genes have been reported in various cancers. Some studies have shown that sine oculis homeobox 1 (*SIX1*) is a key transcription factor in tumorigenesis and has an important role in tumorigenesis [30]. For example, *SIX1* predicts poor prognosis and promotes progression of non-small lung cancers by activating the Notch signaling pathway [31]. *SIX1* is also involved in the regulatory axis of Circular RNA and microRNAs to promote cancer proliferation, migration and invasion [32]. In particular, it has been found to be associated with the EMT process in gynecological tumors [30]. A few studies have confirmed the association of *SIX1* as a malignant factor with endometrial carcinogenesis and development, which echoes the results of the present study [33, 34]. Sirtuin 2 (*SIRT2)* is a histone deacetylase that depends on nicotinamide adenine dinucleotide (NAD +) [35]. The role played by *SIRT2* in cancer is still controversial. *SIRT2* can play a pro-cancer role in gastric cancer [36], but can act as a protective factor in colorectal [37, 38] and cervical cancers [39]. A study has shown that *SIRT2* is highly expressed in EC and can promote cancer proliferation and metastasis by regulating the *RAS/ERK* pathway [40]. However, it has also been said that *SIRT2* is at low expression in EC compared to non-tumorigenic endometrium [41]. In our study, we found that there was no notably difference of *SIRT2* expression both in mRNA and protein level in EC tissues. This may be due to the relatively small sample size, which may limit the generalizability and statistical power of this finding. However, ectopic expression of SIRT2 could promote EC cells metastasis, which was in agreement with previous studies [42]. Based on the controversial roles of *SIRT2* in EC risk and progression, we speculate that the downregulation of *SIRT2* may be an adaptive strategy of tumor cells to evade immune surveillance and thus promote cell proliferation and metastasis [43]. Nevertheless, the precise regulatory mechanisms of *SIRT2* still need to be further verified in independent investigations.

Cyclin dependent kinase inhibitor 2A (*CDKN2A*), also known as P16, is a tumor suppressor gene that can induce cancer cell senescence. Enhancing P16 activity by chemotherapy drugs is a valuable therapeutic strategy for cancer treatment [44]. A large number of studies have shown that *CDKN2A* is involved in poor prognosis in a variety of cancers, including hepatocellular carcinoma [45], cervical cancer [46], ovarian cancer [47] and EC [48]. However, the exact mechanism by which *CDKN2A* causes poor prognosis in cancer is still unclear. Progesterone receptor (*PGR*) is ligand-dependent transcription factors that belongs to the nuclear receptor superfamily. It has been reported to be involved in the growth, development and function of female reproductive tract tissues [49]. *PGR* exhibits high polymorphic, with multiple SNPs having been identified [50]. As a result, it is widely utilized as a biomarker in both breast cancer [51] and EC [52–54]. Despite the fact that most patients with EC exhibit decreased expression of progesterone receptors, there have only been a few mechanistic experiments targeting *PGR* and cancer cells [54].

Our survival analysis indicated that *SIX1, SIRT2,* and *CDKN2A* have a harmful role in EC prognosis, whereas the opposite is true for *PGR*. Afterward, we constructed a risk signature based on the these four EMT-related genes to explore the relationship between EC and risk score. Kaplan–Meier curve and Cox regression analysis showed that patients in the high-risk group had a significantly worse survival. ROC curves validated the reliability of determining risk scores of EC patients by four independent prognostic factors. We also constructed a nomogram integrating risk score and several clinical characteristics that could predict EC clinical outcomes well. By predicting the prognostic risk scores, our 4-gene signature may also provide a basis for risk stratification of EC patients, which may inform the selection of different treatment strategies according to the patient’s risk level. To explore the underlying mechanisms of the 4 EMT-related genes, we constructed co-expression networks using several strategies and performed functional enrichment analysis. We found that the co-expressed genes of the four EMT-related genes were mainly present with the function of extracellular matrix tissue and pathway of mesenchymal development. This suggests that these four genes are indeed associated with EMT and may be involved in mechanisms related to metastasis. We initially verified the effect of these four genes on the metastasis ability of EC cells through cellular experiments. This makes our model more convincing. Combined with previous reported evidence [55], our findings suggest that these four genes may be involved in the ecological adaptation of EC cells to the tumor microenvironment and macroenvironment, and may affect their survival and dissemination. We believe that our study not only provides a novel prognostic biomarker for EC patients, but also contributes to the understanding of cancer and EMT from an ecological perspective.

This study utilized a large dataset from TCGA database for bioinformatic analysis, and the meaningful results were further validated in our independent samples and series of functional experiments, increasing the credibility of the study results. In addition, the nomogram integrating the risk signature and clinical characteristics exhibited robust validity and performance at predicting EC overall survival, which may provide a useful tool for clinicians. Besides, in order to further prove the predictive performance of the ROC curve in our model, we compare three recently published articles on the signatures of the prognostic model in EC [56, 57]. Based on the same TCGA patient cohort, we found that in this model, the AUC of 5 years-OS for our signatures is 0.793, which is significantly higher than that of other existing EMT-related signatures. Meanwhile, there may be some possible limitations in this study. Firstly, this study is based on retrospective data analysis, which may be subject to selection bias and confounding factors. Further validation in independent cohorts is necessary to confirm the reliability of the risk signature. Secondly, in our analysis, we used a f a Benjamini–Hochberg adjusted p-value of 0.05 to identify significant DEGs. A more stringent filtering criteria, such as a higher fold-change or a lower *P* value threshold, would generate a shorter list of DEGs, but may also exclude genes that could be biologically relevant. Our finding showing a concomitant deregulation of almost 80% of the EMT-related genes suggests a complex regulatory network rather than a single molecular mechanism driving EMT. Thirdly, our nomogram does not include treatment information, which may affect the survival of EC patients. The TCGA data contains 31 different therapeutic approaches, which makes it difficult to analyze the effect of each treatment on survival. Moreover, some treatments may be confounded by other factors such as disease stage, tumor grade, or patient comorbidities. Therefore, we decided to focus on developing a nomogram based on clinical and molecular features that are readily available at diagnosis and can provide a general prognosis for EC patients. However, we acknowledge that incorporating treatment information into our nomogram would be valuable for predicting treatment outcomes and optimizing treatment choices. Future studies should collect more standardized and detailed treatment data for EC patients and incorporate them into prognostic models. Lastly, while the cellular assays provided preliminary evidence regarding the functional role of the identified genes in EC progression, further investigations are needed to elucidate the biological mechanisms underlying their prognostic value and potential therapeutic applications.

## Conclusions

In conclusion, our study identified a novel four-gene EMT-related signature that could serve as a potential biomarker for EC prognosis. The finding that a large proportion of EMT-related genes are differentially expressed in EC highlights the complexity of the regulatory networks involved in EMT and cancer progression. Further studies are needed to validate and extend our findings and to explore the functional roles and mechanisms of the DEGs identified in this study.

### Supplementary Information

**Additional file 1: Figure S1. **The associations between 540 EMT-related DEGs and EC progression.

**Additional file 2: Figure S2. **The associations between 4 EMT-related genes and EC progression-free interval (PFI).

**Additional file 3: Figure S3. **The associations between 4 EMT-related genes and EC disease-free interval (DFI).

**Additional file 4: Figure S4. **The association between risk scores and EC PFI and DFI. A. PFI. B. DFI.

**Additional file 5: Figure S5. **The distribution and median value of the risk scores in the UCEC-TCGA cohort.

**Additional file 6: Figure S6.  **Co-expression networks of the 4 EMT-related genes using STRING database. A. Constructing co-expression networks of the 4 EMT-related genes using STRING database. B. GO enrichment results. C. KEGG enrichment results.

**Additional file 7: Figure S7.  **Co-expression networks of the 4 EMT-related genes from cBioportal.

**Additional file 8: Figure S8.  **Enrichment analyses using networks constructed of the 4 EMT-related genes by cBioportal. A. GO enrichment results. B. KEGG enrichment results.

**Additional file 9. **

**Additional file 10: Table S1.**Clinicopathological characteristics and survival status of the patients from TCGA. **Table S2. **The characteristics of selected GEO datasets of EC. **Table S3. **The clinicopathological characteristics in patients with endometrial cancer. **Table S4. **siRNA sequence list. **Table S5. **PCR primer sequence list. **Table S6. **The results of LASSO-COX regression model. 

## Data Availability

The datasets analyzed during the current study are available in the UCSC Xena public platform (https://xenabrowser.net/datapages/?dataset=TCGA-UCEC.htseq_counts.tsv&host=https%3A%2F%2Fgdc.xenahubs.net&removeHub=https%3A%2F%2Fxena.treehouse.gi.ucsc.edu%3A443. Access to 2022–4-1), GeneCards (https://www.genecards.org/Search/Keyword?queryString=epithelial-to-mesenchymal%20transition. Access to 2022–4-28) and GEO repository (http://www.ncbi.nlm.nih.gov/geo/, accession number: GSE56087, GSE106191, GSE17025, GSE115810, GSE36389 and GSE63678. Access to 2022–4-1). The immunohistochemical figures generated during the current study are available in The Human Protein Atlas repository (SIRT2:https://www.proteinatlas.org/ENSG00000068903-SIRT2/tissue/endometrium; https://www.proteinatlas.org/ENSG00000068903-SIRT2/pathology/endometrial+cancer; SIX1:https://www.proteinatlas.org/ENSG00000126778-SIX1/tissue/endometrium; https://www.proteinatlas.org/ENSG00000126778-SIX1/pathology/endometrial+cancer; CDKN2A:https://www.proteinatlas.org/ENSG00000147889-CDKN2A/tissue/endometrium; https://www.proteinatlas.org/ENSG00000147889-CDKN2A/pathology/endometrial+cancer; PGR: https://www.proteinatlas.org/ENSG00000082175-PGR/tissue/endometrium; https://www.proteinatlas.org/ENSG00000082175-PGR/pathology/endometrial+cancer. Access to 2022–5-12.).
