# Supplementary figures and images for: An EMT-related genes signature as a prognostic biomarker for patients with endometrial cancer
Source: BMC Cancer. 2023 Sep 18;23:879. doi: 10.1186/s12885-023-11358-4 (PMC10506329; doi:10.1186/s12885-023-11358-4)

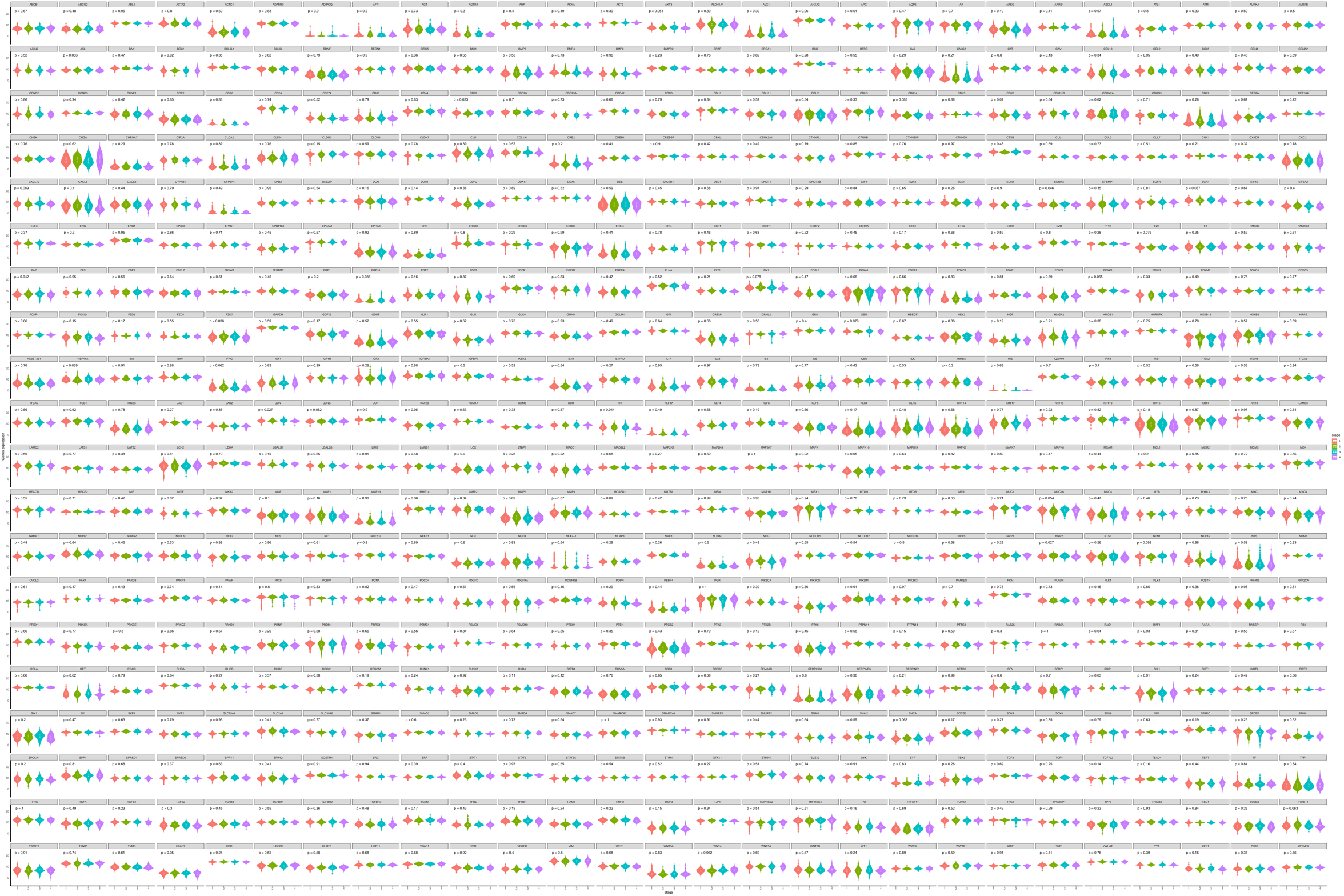

Supplement: Supplementary file 1 — Additional file 1: Figure S1. The associations between 540 EMT-related DEGs and EC progression. [file 12885_2023_11358_MOESM1_ESM.pdf]

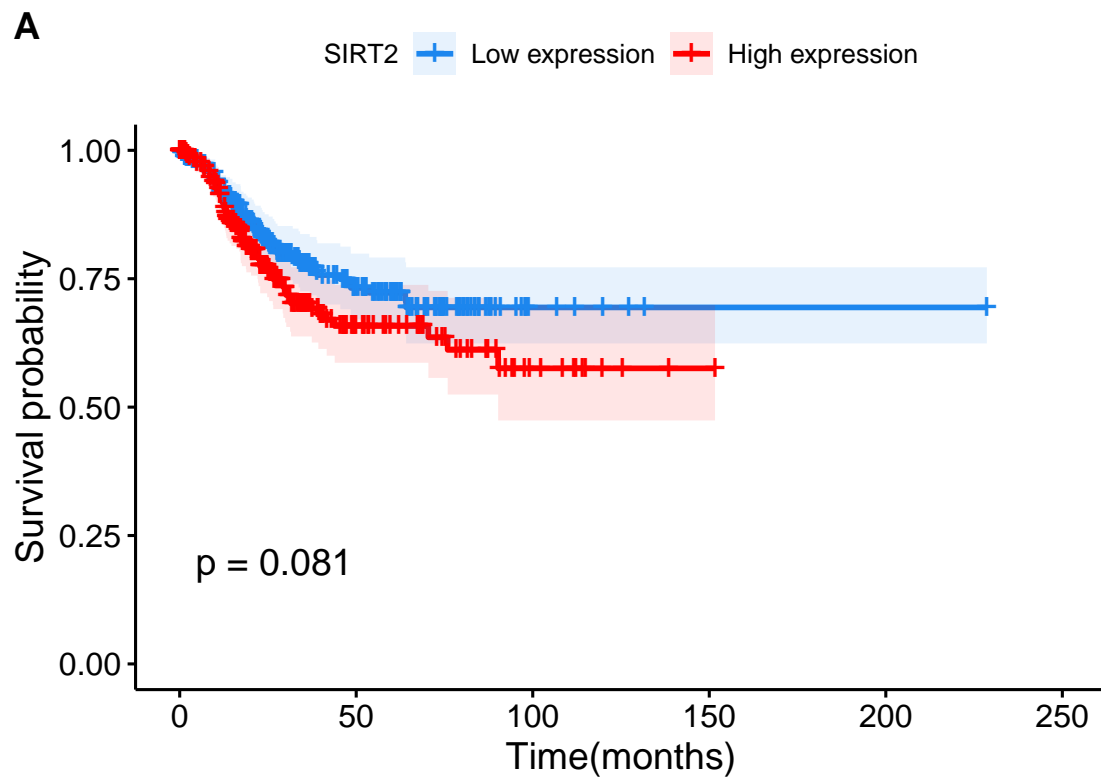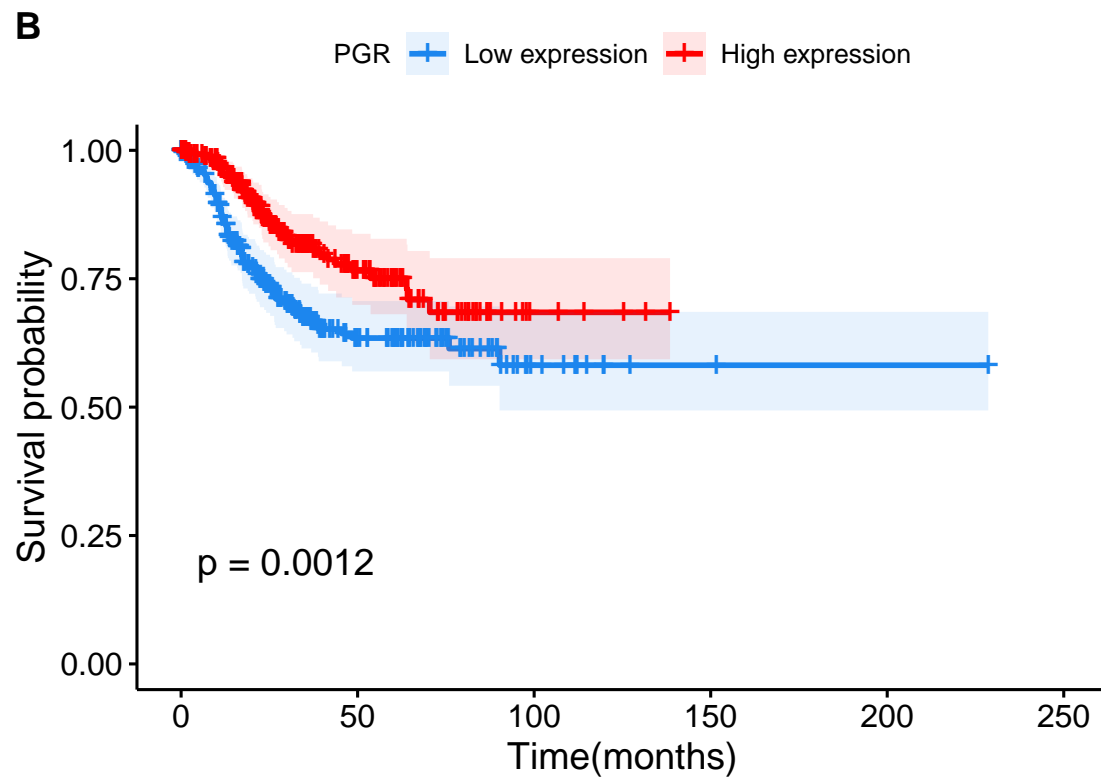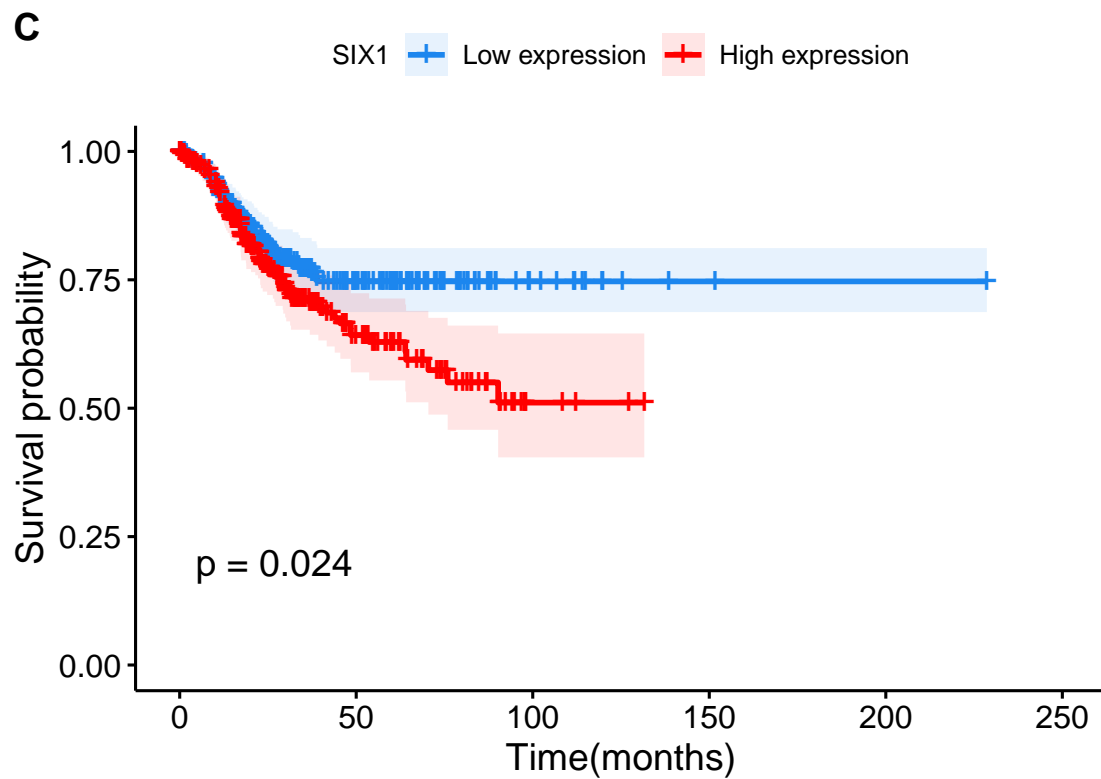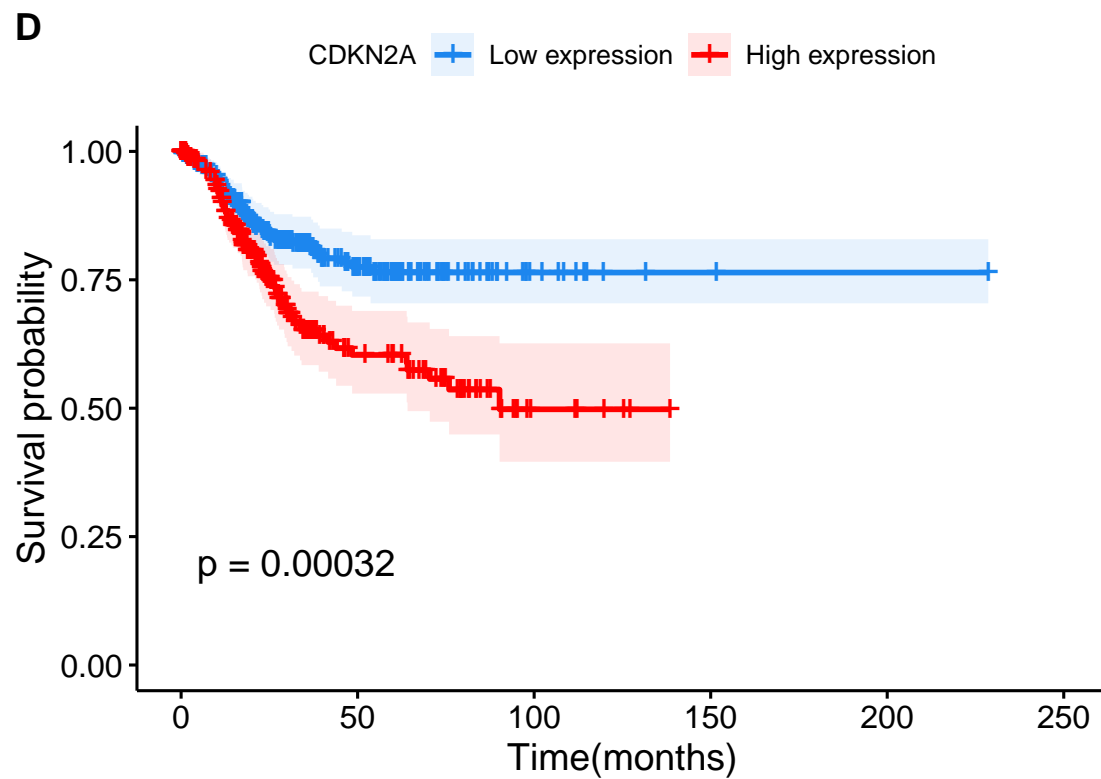

Supplement: Supplementary file 2 — Additional file 2: Figure S2. The associations between 4 EMT-related genes and EC progression-free interval (PFI). [file 12885_2023_11358_MOESM2_ESM.pdf]

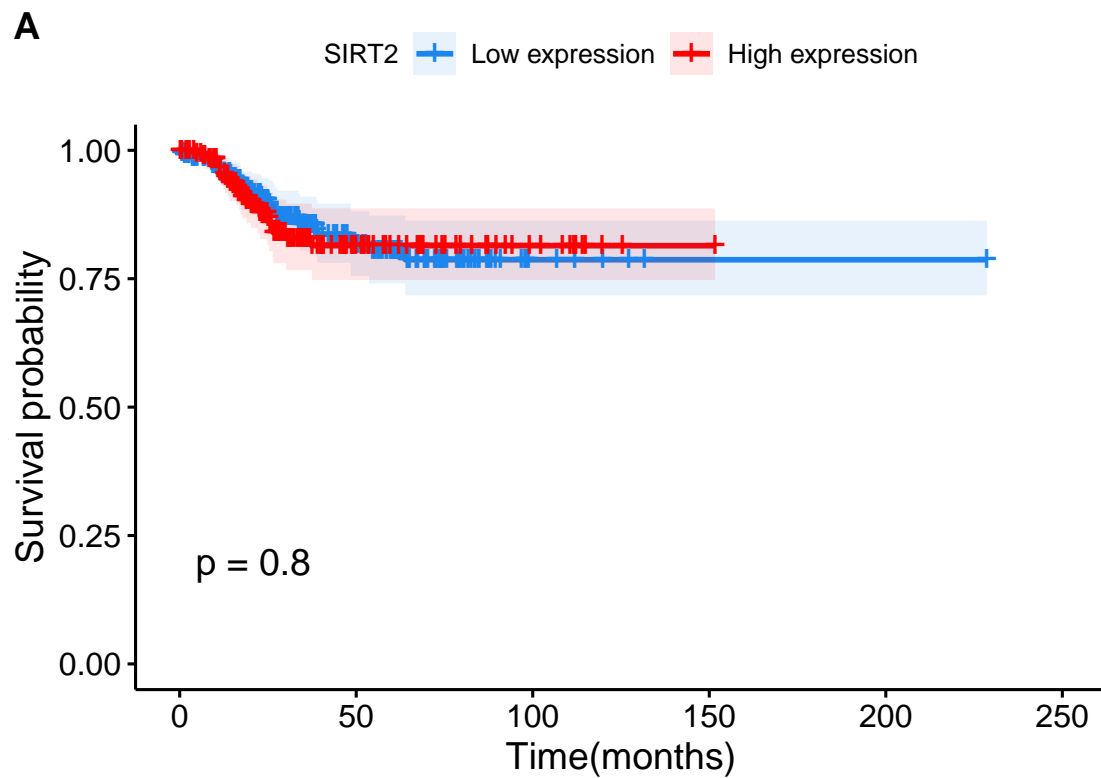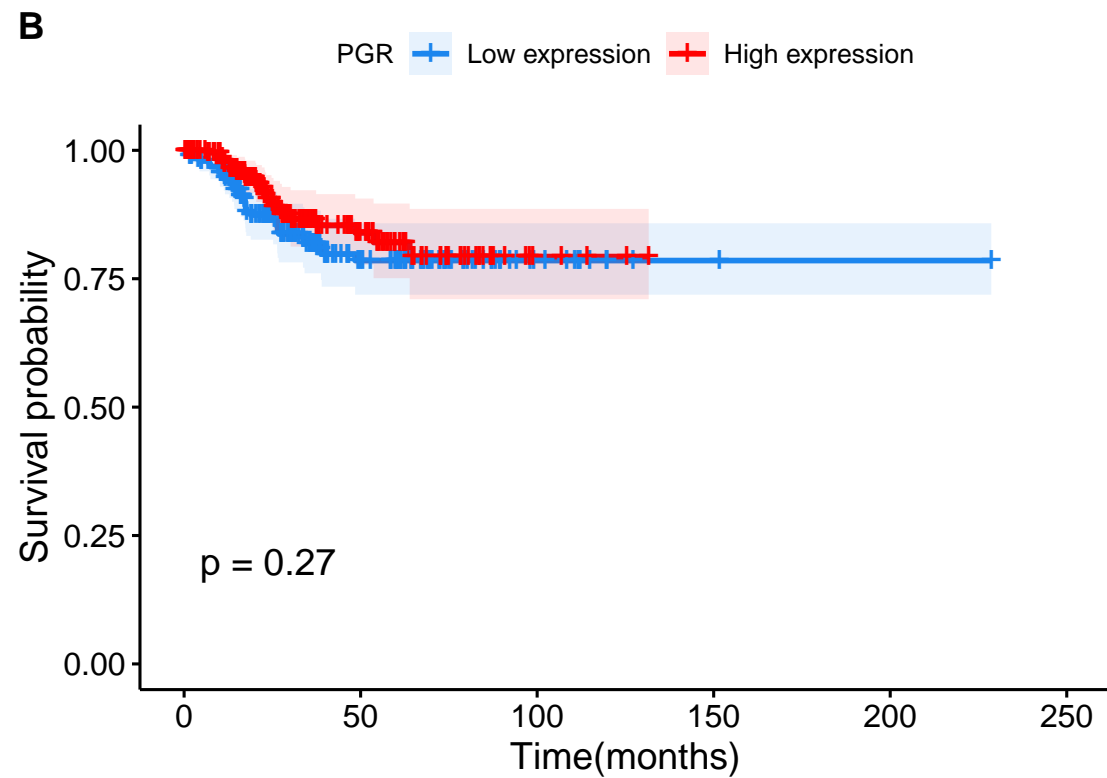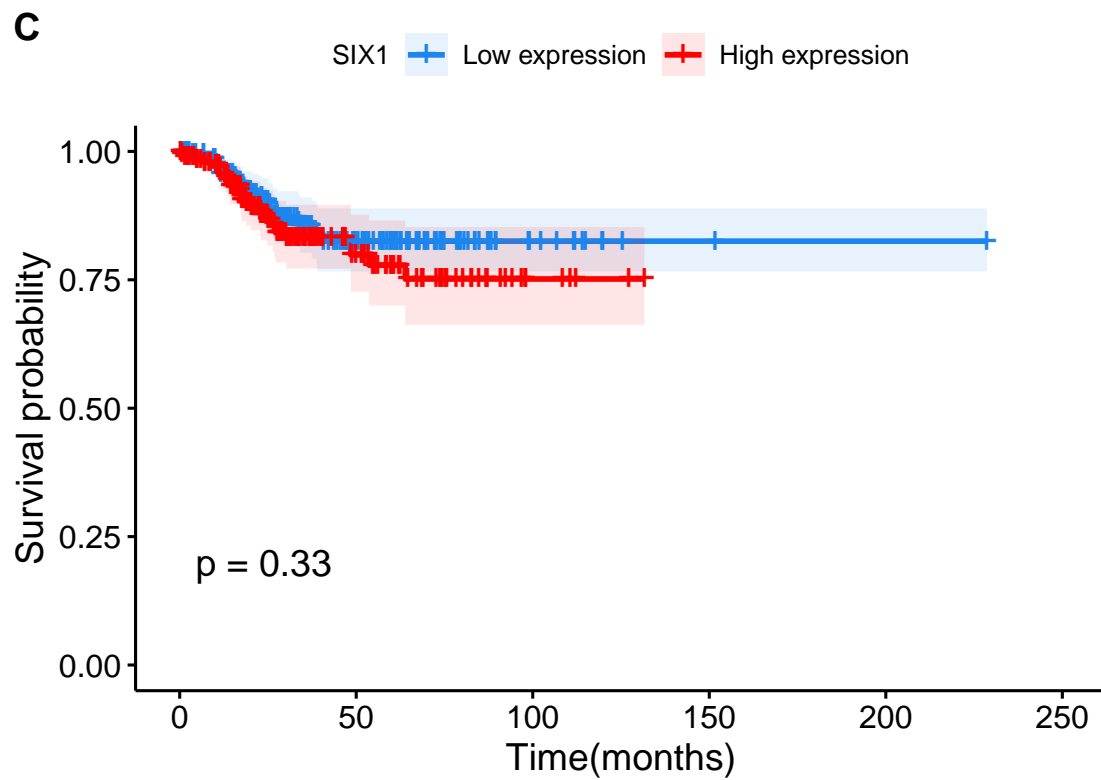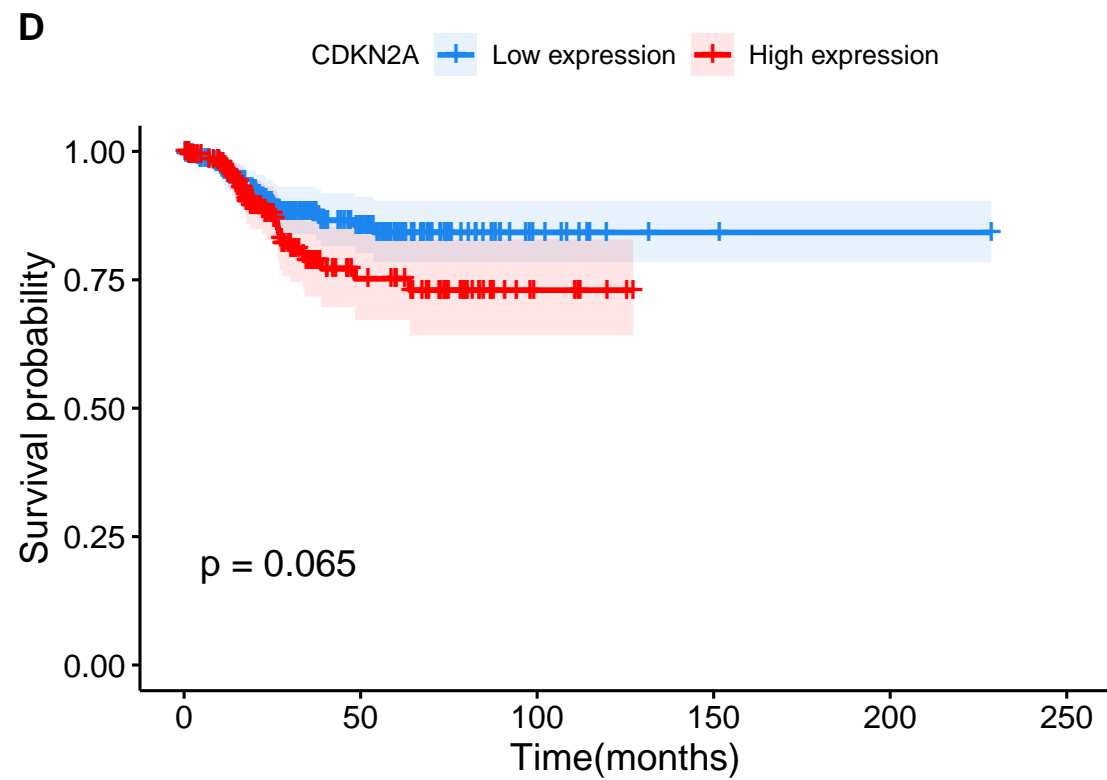

Supplement: Supplementary file 3 — Additional file 3: Figure S3. The associations between 4 EMT-related genes and EC disease-free interval (DFI). [file 12885_2023_11358_MOESM3_ESM.pdf]

**A**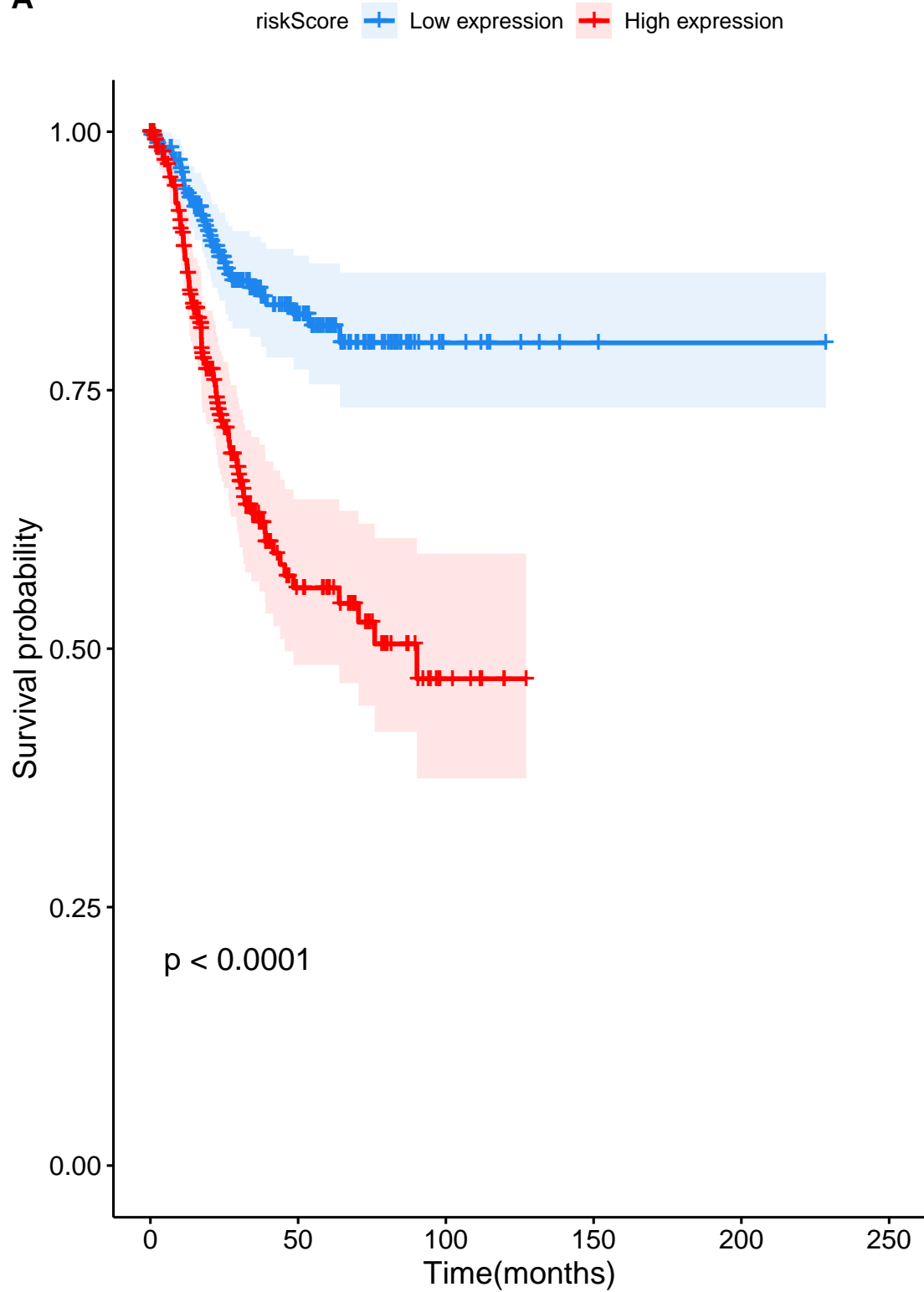**B**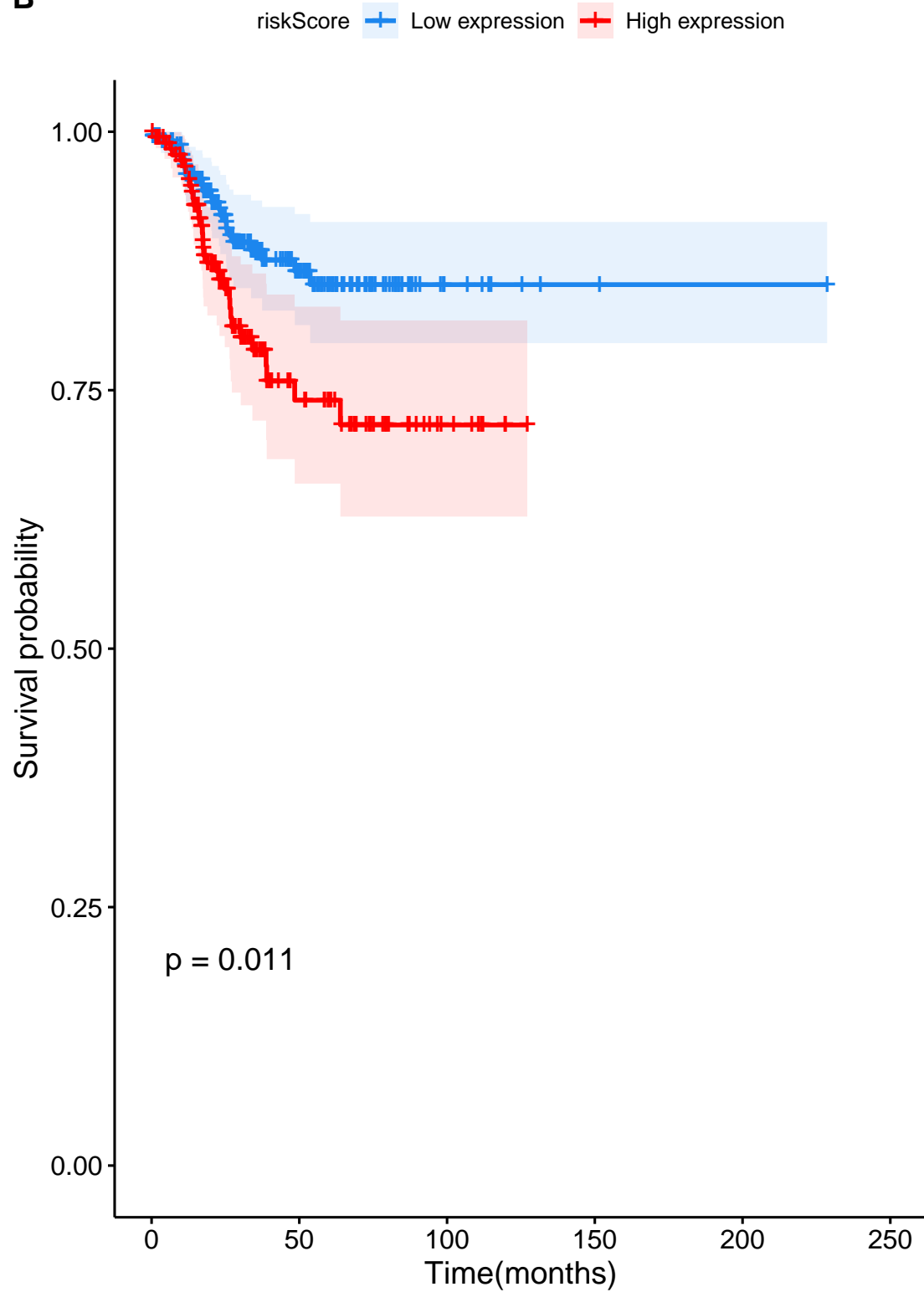

Supplement: Supplementary file 4 — Additional file 4: Figure S4. The association between risk scores and EC PFI and DFI. A. PFI. B. DFI. [file 12885_2023_11358_MOESM4_ESM.pdf]

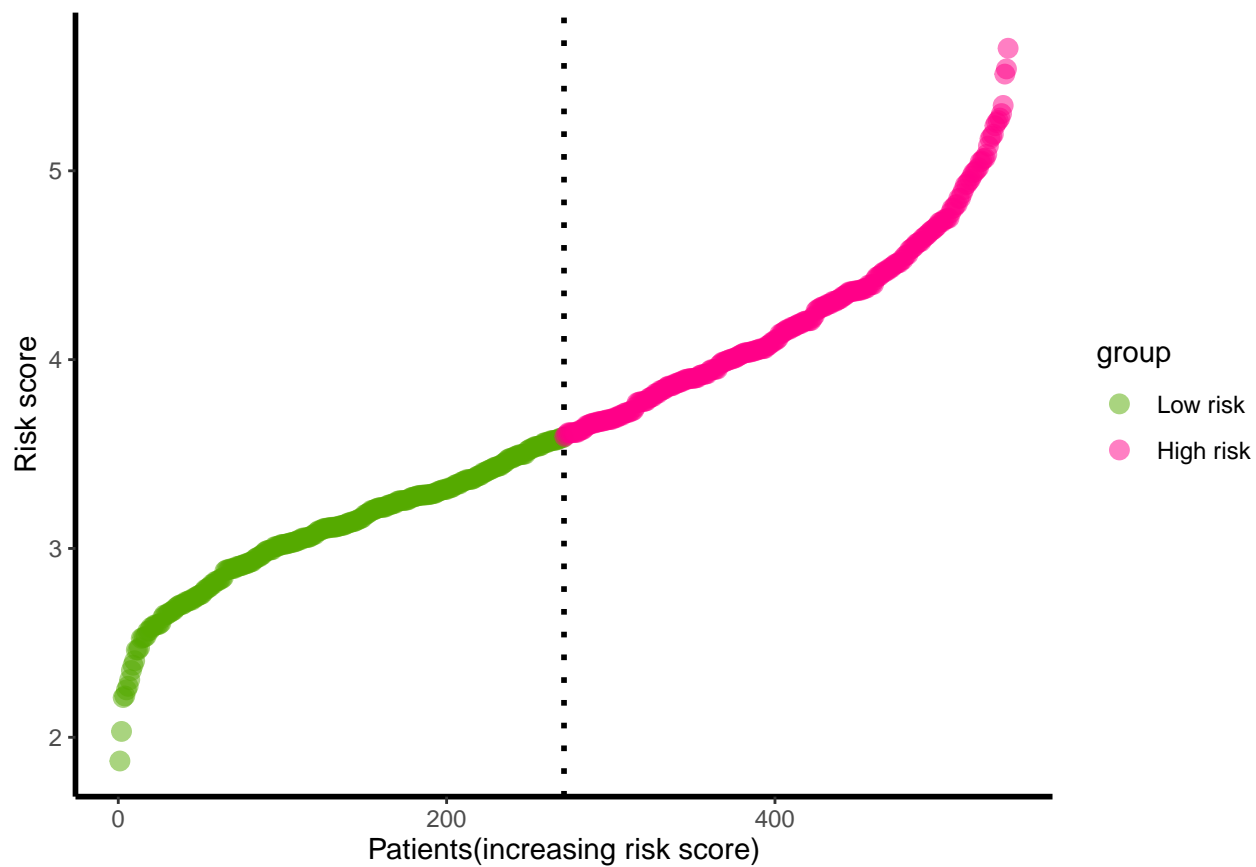

Supplement: Supplementary file 5 — Additional file 5: Figure S5. The distribution and median value of the risk scores in the UCEC-TCGA cohort. [file 12885_2023_11358_MOESM5_ESM.pdf]

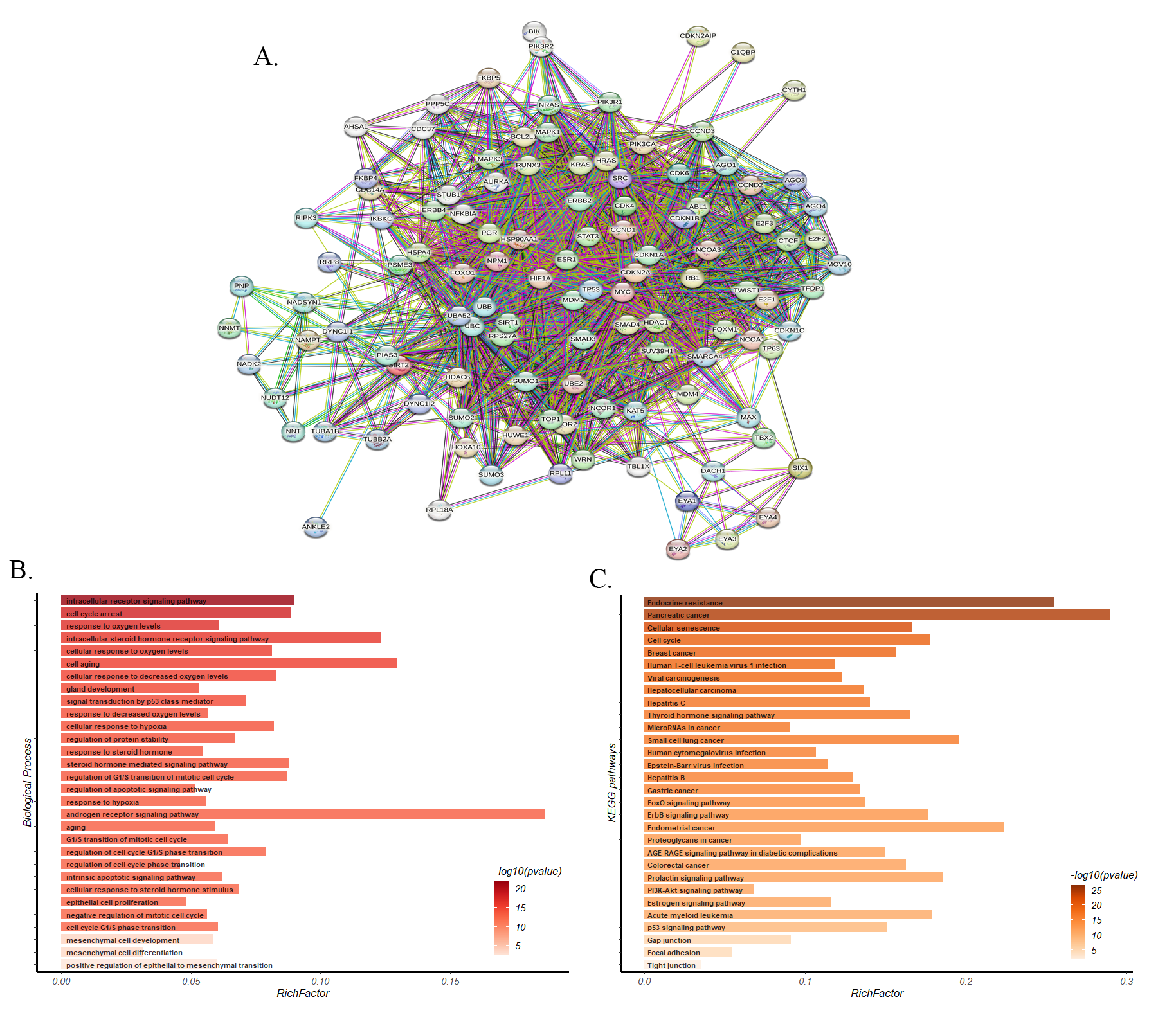

Supplement: Supplementary file 6 — Additional file 6: Figure S6. Co-expression networks of the 4 EMT-related genes using STRING database. A. Constructing co-expression networks of the 4 EMT-related genes using STRING database. B. GO enrichment results. C. KEGG enrichment results. [file 12885_2023_11358_MOESM6_ESM.tif]

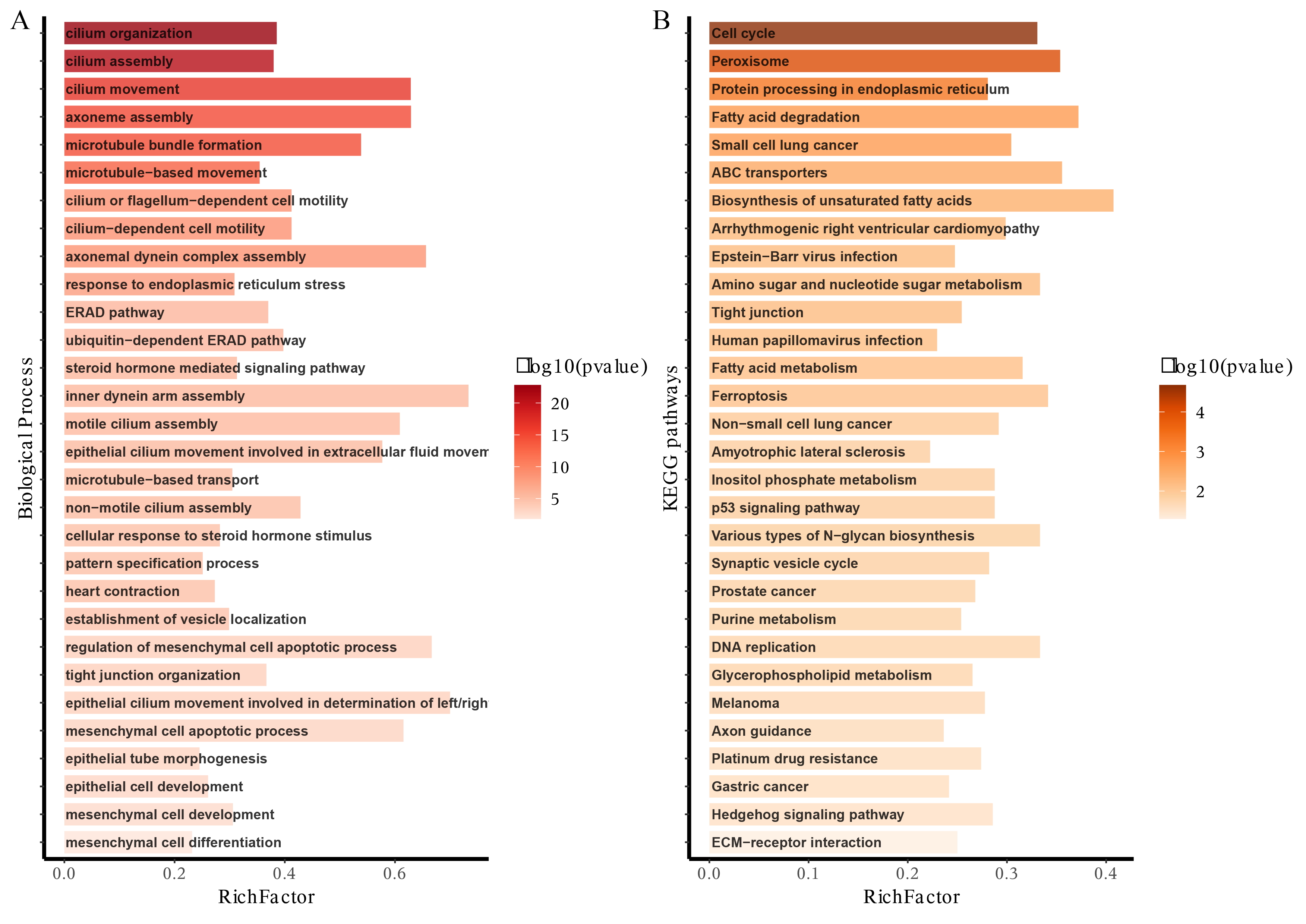

Supplement: Supplementary file 8 — Additional file 8: Figure S8. Enrichment analyses using networks constructed of the 4 EMT-related genes by cBioportal. A. GO enrichment results. B. KEGG enrichment results. [file 12885_2023_11358_MOESM8_ESM.tif]

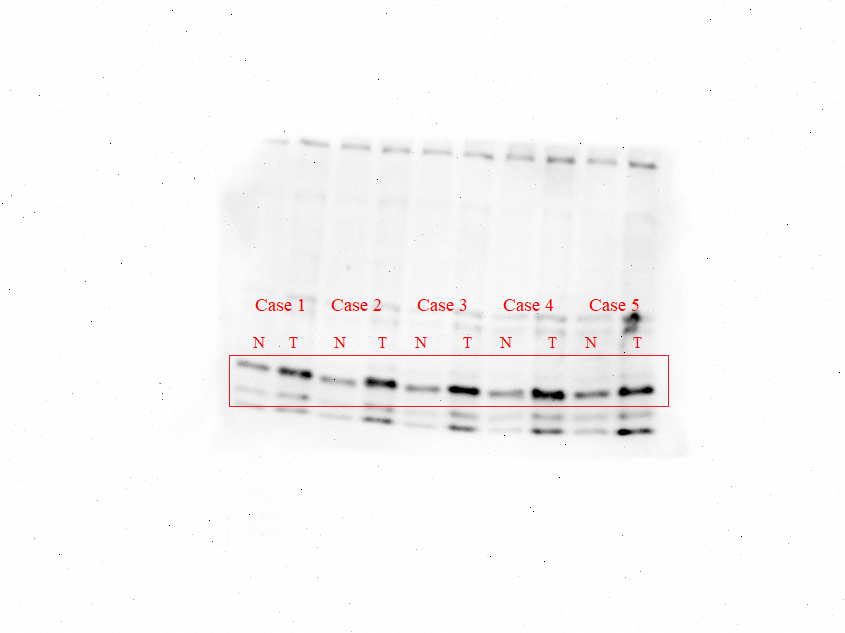

Supplement: Supplementary file 9 — Additional file 9. [file 12885_2023_11358_MOESM9_ESM.zip › Original files for CDKN2A in Figure 3D.TIF]

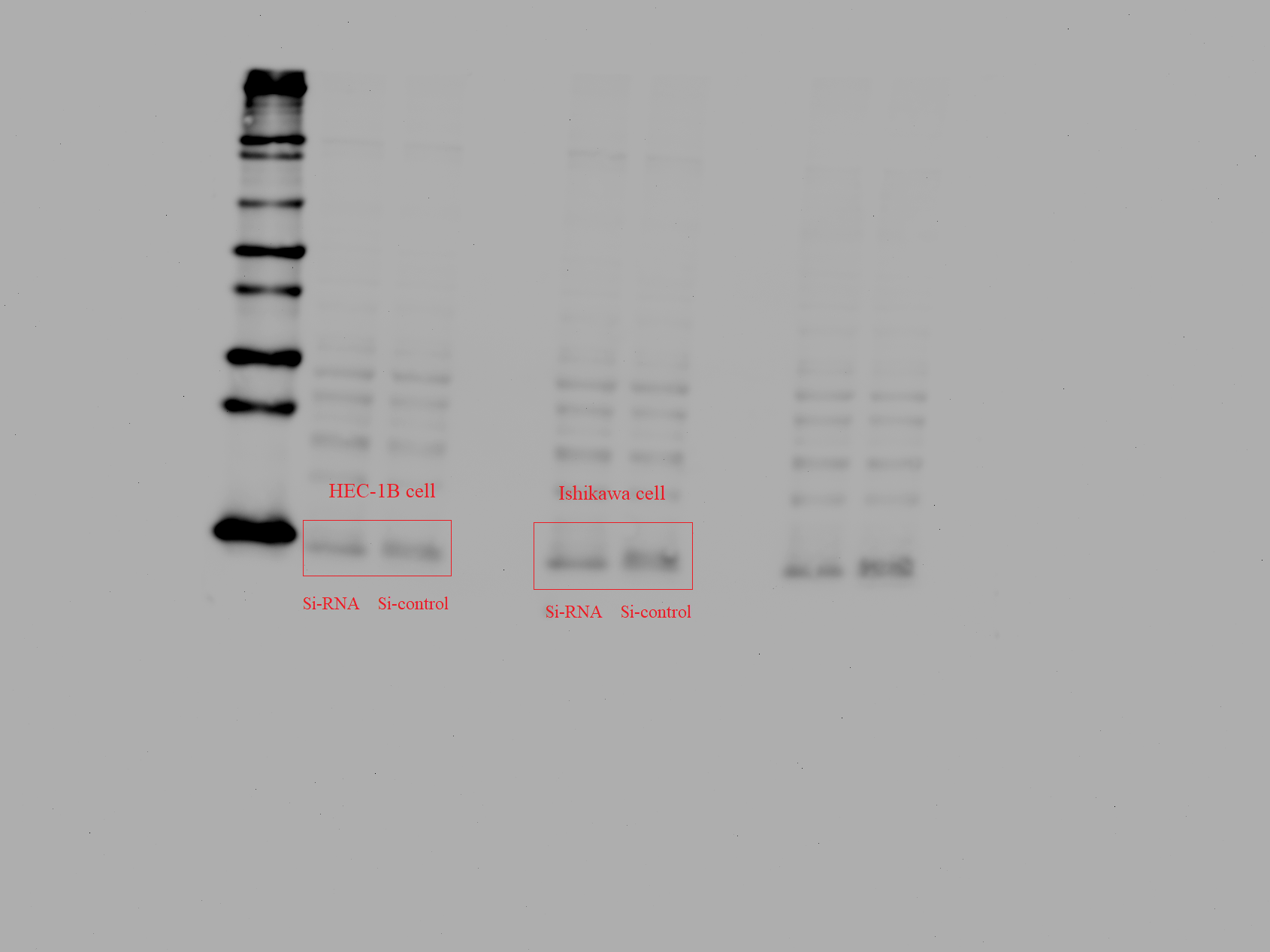

Supplement: Supplementary file 9 — Additional file 9. [file 12885_2023_11358_MOESM9_ESM.zip › Original files for CDKN2A in Figure 8B.TIF]

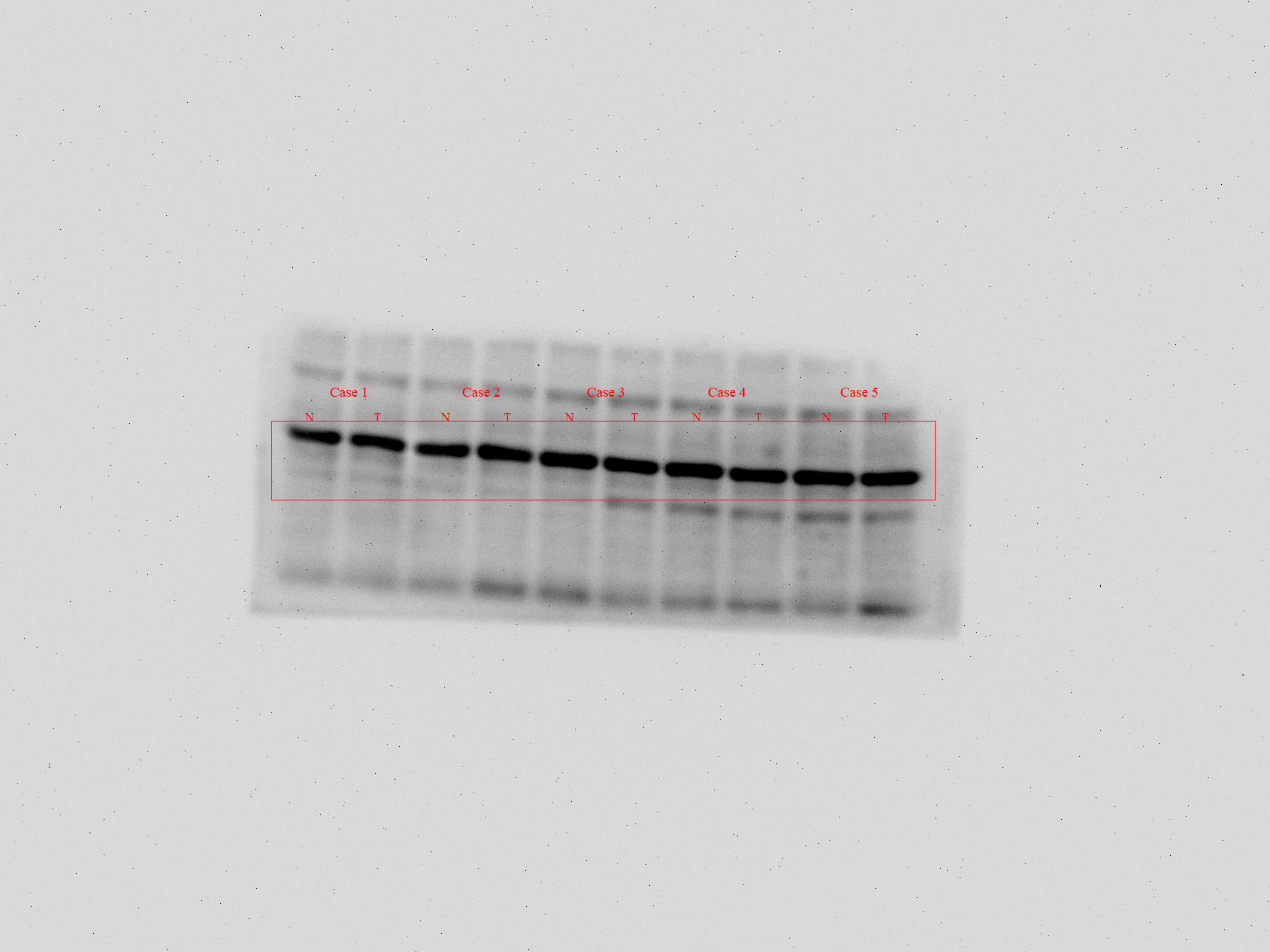

Supplement: Supplementary file 9 — Additional file 9. [file 12885_2023_11358_MOESM9_ESM.zip › Original files for GAPDH in Figure 3D.TIF]

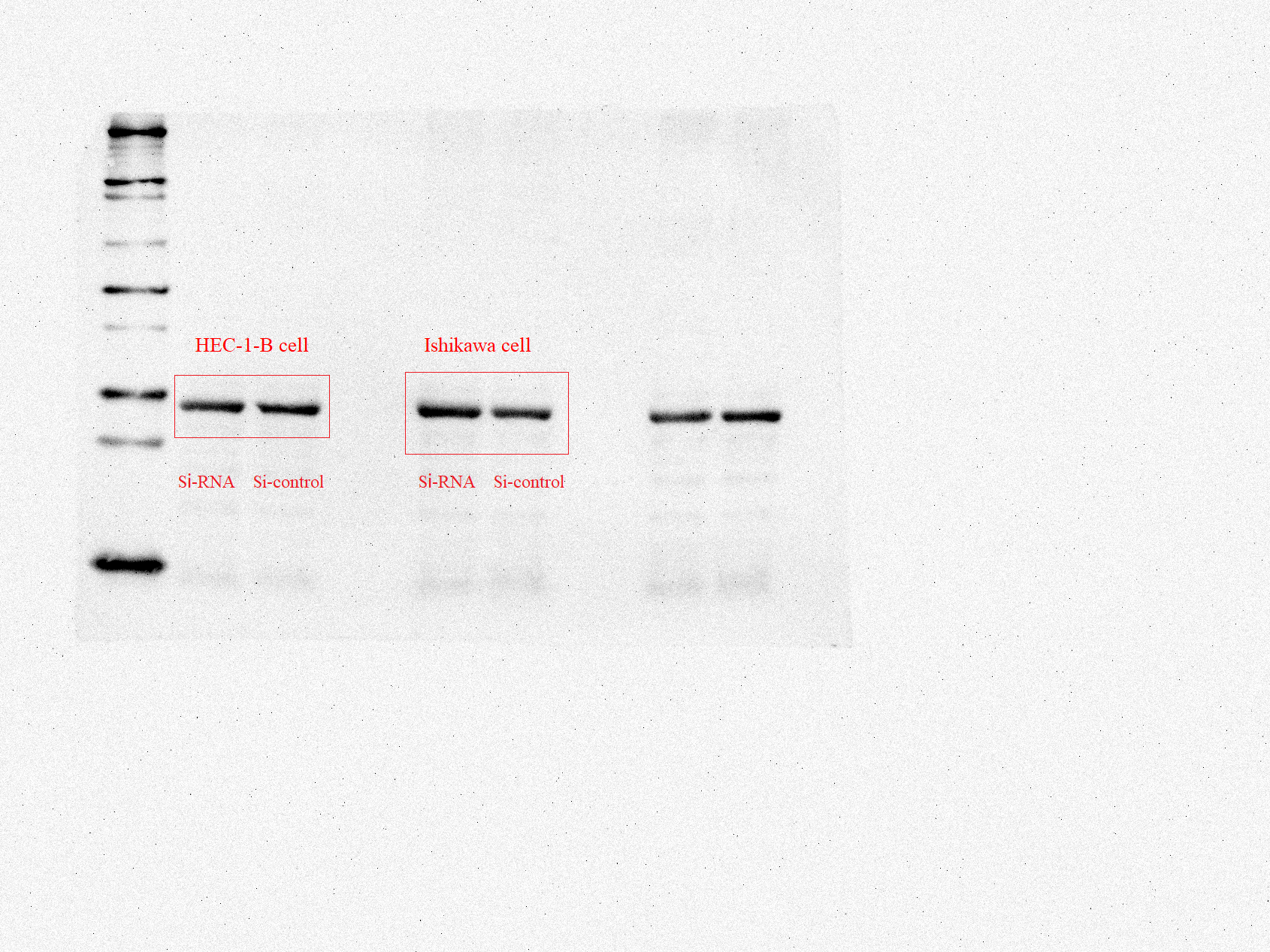

Supplement: Supplementary file 9 — Additional file 9. [file 12885_2023_11358_MOESM9_ESM.zip › Original files for GAPDH in Figure 8B.TIF]

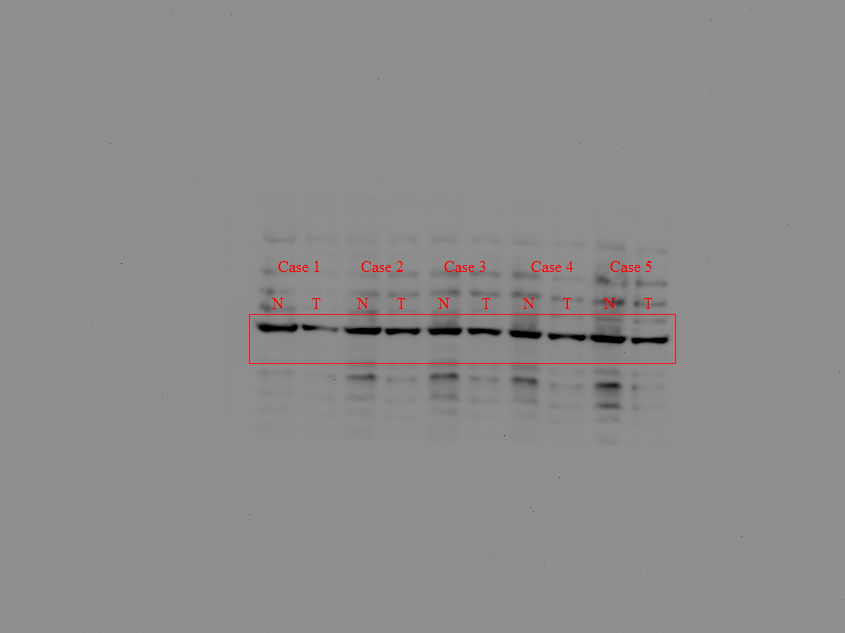

Supplement: Supplementary file 9 — Additional file 9. [file 12885_2023_11358_MOESM9_ESM.zip › Original files for PGR in Figure 3D.TIF]

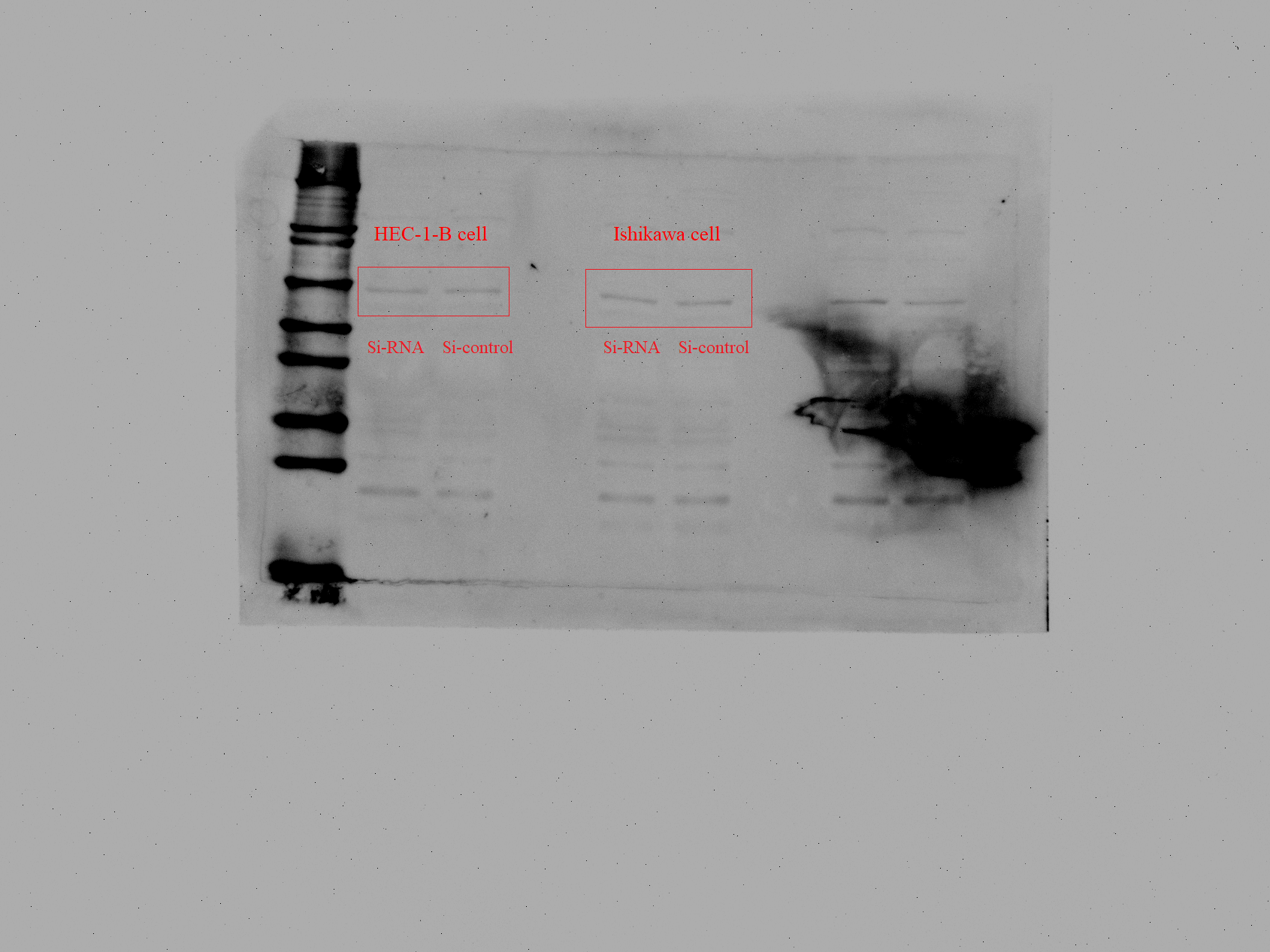

Supplement: Supplementary file 9 — Additional file 9. [file 12885_2023_11358_MOESM9_ESM.zip › Original files for PGR in Figure 8B .TIF]

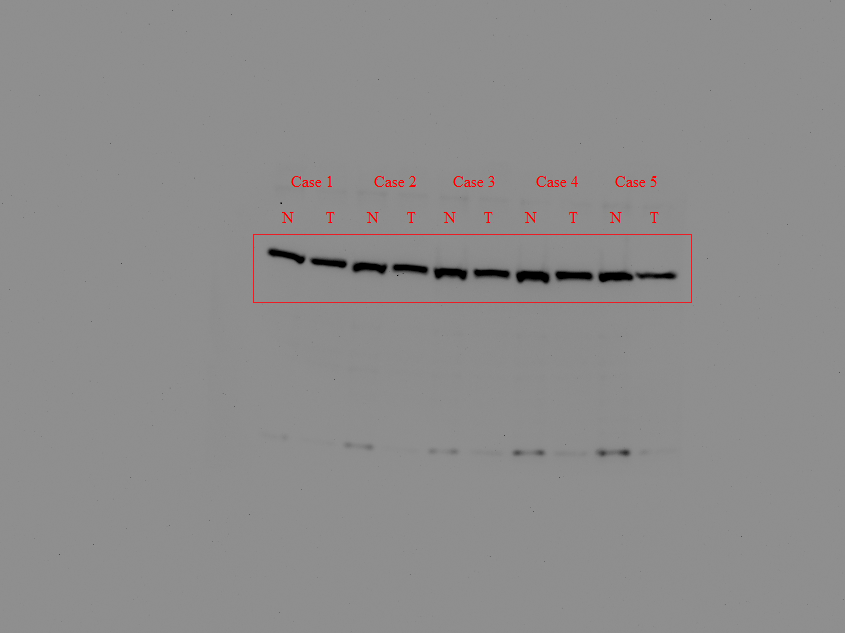

Supplement: Supplementary file 9 — Additional file 9. [file 12885_2023_11358_MOESM9_ESM.zip › Original files for SIRT2 in Figure 3D.TIF]

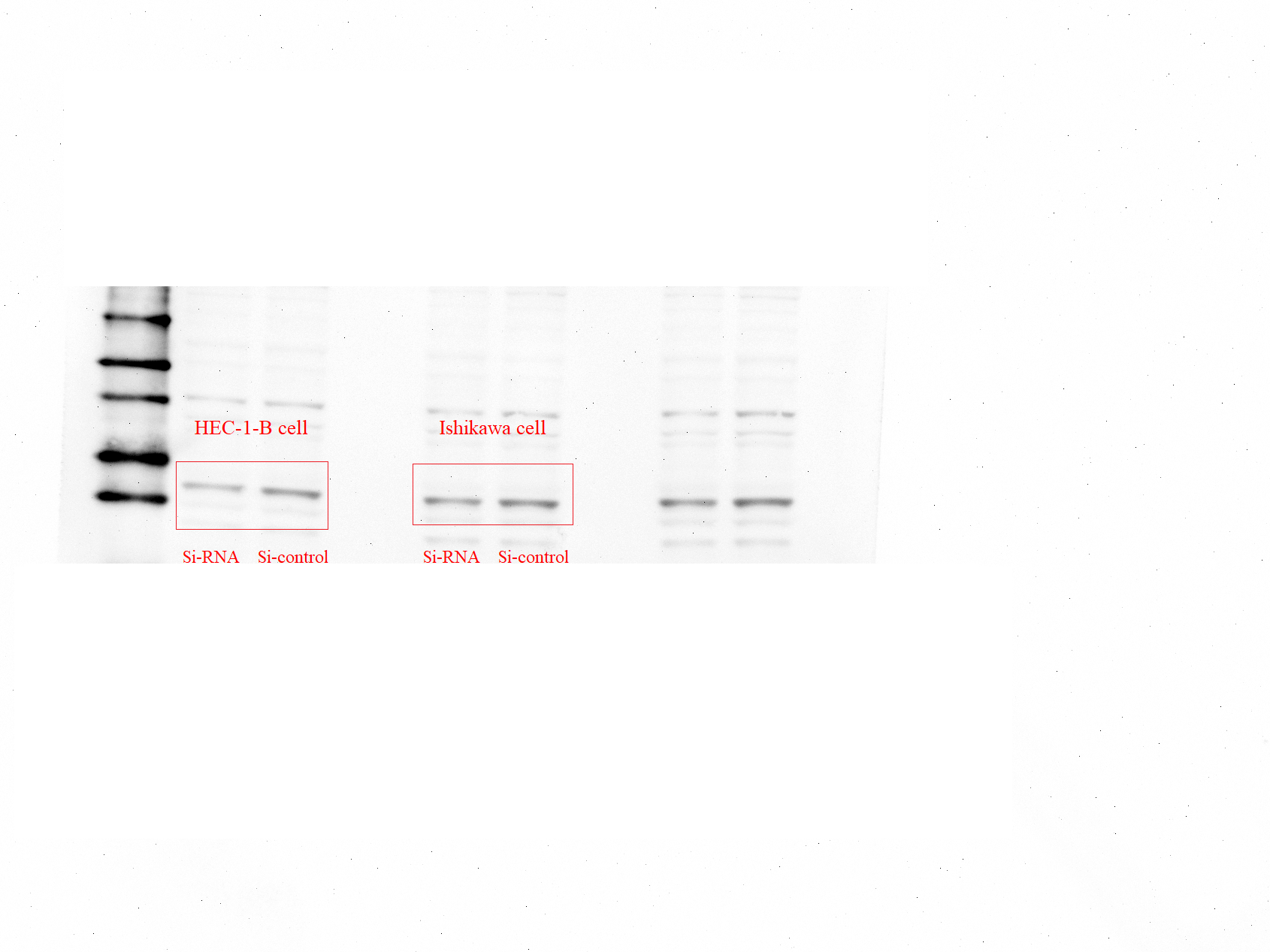

Supplement: Supplementary file 9 — Additional file 9. [file 12885_2023_11358_MOESM9_ESM.zip › Original files for SIRT2 in Figure 8B.TIF]

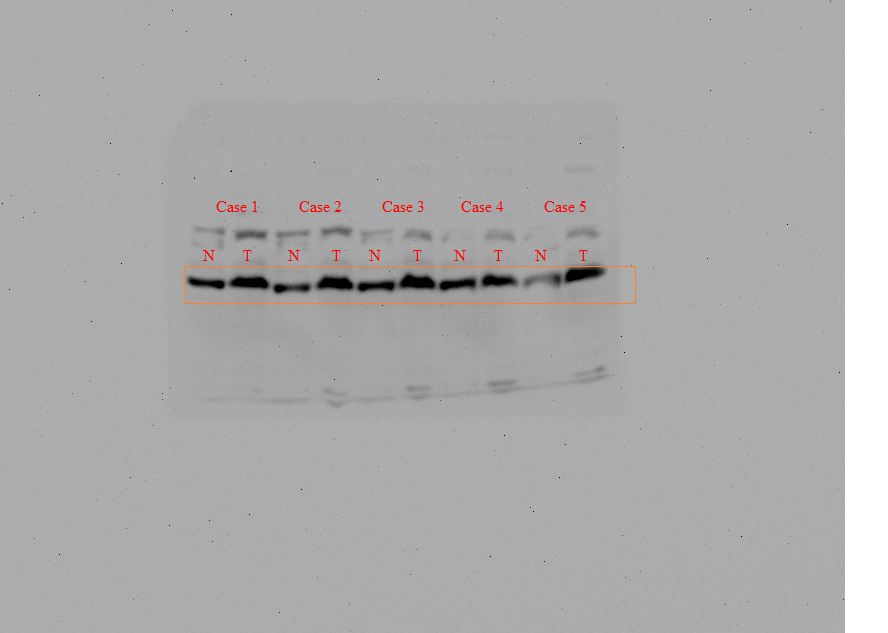

Supplement: Supplementary file 9 — Additional file 9. [file 12885_2023_11358_MOESM9_ESM.zip › Original files for SIX1 in Figure 3D.TIF]

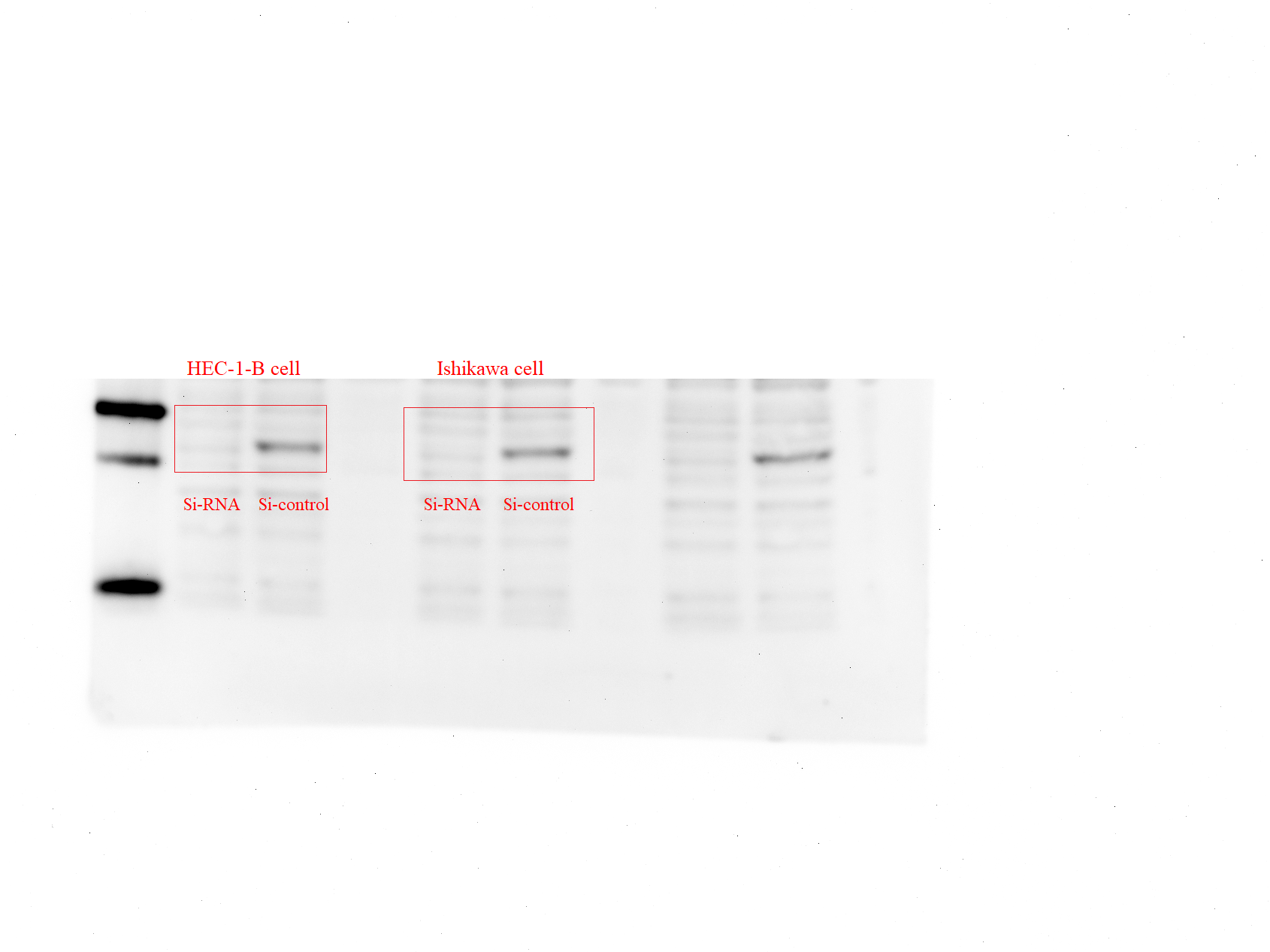

Supplement: Supplementary file 9 — Additional file 9. [file 12885_2023_11358_MOESM9_ESM.zip › Original files for SIX1 in Figure 8B.TIF]
