# Supplementary material for: An EMT-related genes signature as a prognostic biomarker for patients with endometrial cancer
Source: BMC Cancer. 2023 Sep 18;23:879. doi: 10.1186/s12885-023-11358-4 (PMC10506329; doi:10.1186/s12885-023-11358-4)
Supplement: Supplementary file 10 — Additional file 10: Table S1.Clinicopathological characteristics and survival status of the patients from TCGA. Table S2. The characteristics of selected GEO datasets of EC. Table S3. The clinicopathological characteristics in patients with endometrial cancer. Table S4. siRNA sequence list. Table S5. PCR primer sequence list. Table S6. The results of LASSO-COX regression model. [file 12885_2023_11358_MOESM10_ESM.docx]

**Supplementary Tables**

**Table S1: Clinicopathological characteristics and survival status of the patients from TCGA**

| Characteristics | Endometrial cancer patients (N=542) | |
| --- | --- | --- |
| Race,n(%) | White | 371(68.45) |
|  | Asian | 20(3.69) |
|  | Black or African American | 106(19.56) |
|  | American Indian or Alaska Native | 4(0.74) |
|  | Native Hawaiian or Other Pacific Islander | 9(1.66) |
|  | Unknown | 32(5.90) |
| Age,Years | 63.99±11.12 | |
| Grade,n(%) | 1 | 98(18.08) |
|  | 2 | 120(22.14) |
|  | 3 | 324(59.78) |
| Stage,n(%) | Ⅰ | 338(62.36) |
|  | Ⅱ | 52(9.59) |
|  | Ⅲ | 122(22.51) |
|  | Ⅳ | 30(5.54) |
| Histological Type,n(%) | Endometrioid endometrial adenocarcinoma | 406(74.91) |
|  | Serous endometrial adenocarcinoma | 114(21.03) |
|  | Mixed serous and endometrioid | 22(4.06) |
| Survival Status,n(%) | Death | 91(16.79) |
|  | Alive | 451(83.21) |
| Survival Time,Months | 38.05±30.17 | |

**Table S2: The characteristics of selected GEO datasets of EC**

| Datasets | Contributor (year) | Experimental platform | Number of cases  (cancer/control) |
| --- | --- | --- | --- |
| GSE56087 | Zhou Y (2015) | Illumina HiSeq 2000 (Homo sapiens) | 9/9 |
| GSE106191 | Sugiyama Y (2019) | Affymetrix Human Genome U133 Plus 2.0 Array | 64/33 |
| GSE17025 | Risinger JH *et al.* (2011) | Affymetrix Human Genome U133 Plus 2.0 Array | 91/12 |
| GSE115810 | Mazurek U (2018) | Affymetrix Human Genome U133A Array | 24/3 |
| GSE36389 | Mazurek U (2012) | Affymetrix Human Genome U133A Array | 13/7 |
| GSE63678 | Polyzos A (2015) | Affymetrix Human Genome U133A 2.0 Array | 7/5 |

**Table S3.** The clinicopathological characteristics in patients with endometrial cancer.

| **Characteristics** |  | **Guangzhou population** | |
| --- | --- | --- | --- |
|  |  | **N**^a^ | **(%)** |
| **Age(years)** |  |  |  |
| ≤60 |  | 28 | (66.7) |
| >60 |  | 14 | (33.3) |
| **Family history of cancer** |  |  |  |
| Positive |  | 1 | (2.4) |
| Negative |  | 41 | (97.6) |
| **Histologic type** |  |  |  |
| Endometrioid |  | 39 | (92.8) |
| Non-endometrioid |  | 3 | (7.2) |
| **FIGO^b^ stage** |  |  |  |
| I |  | 32 | (76.2) |
| II |  | 6 | (14.3) |
| III |  | 4 | (9.5) |
| **Grade** |  |  |  |
| G1 |  | 16 | (38.1) |
| G2 |  | 19 | (45.2) |
| G3 |  | 7 | (16.7) |

^a^ N, Number of patients

^b^ FIGO, The International Federation of Gynecology and Obstetrics.

**Table S4: siRNA sequence list**

| Gene name | Sense（5'-3'） | Antisense（5'-3'） |
| --- | --- | --- |
| *SIRT2* | GGACUUCCUGCGGAACUUATT | UAAGUUCCGCAGGAAGUCCTT |
| *SIX1* | CCAACAAGCAGAACCAACUTT | AGUUGGUUCUGCUUGUUGGTT |
| *CDKN2A* | CCGUAAAUGUCCAUUUAUATT | UAUAAAUGGACAUUUACGGTT |
| *PGR* | GGAGUUUGUCAAGCUUCAATT | UUGAAGCUUGACAAACUCCTT |
| Negative control | UUCUCCGAACGUGUCACGUTT | ACGUGACACGUUCGGAGAATT |

| Gene name | Forward primer（5'-3'） | Reverse primer（5'-3'） |
| --- | --- | --- |
| *SIRT2* | CACGCAGAACATAGATACCCTG | CAGTGTGATGTGTAGAAGGTGC |
| *SIX1* | CTGCCGTCGTTTGGCTTTAC | GCTCTCGTTCTTGTGCAGGT |
| *CDKN2A* | ATGTCGCACGGTACCTG | GTTGTGGCCCTGTAGGA |
| *PGR* | AAGGAGTTGTGTCGAGCTCA | GTTTCACCATCCCTGCCAAT |
| *β-actin* | GGCGGCACCACCATGTACCCT | AGGGGCCGGACTCGTCATACT |

**Table S5: PCR primer sequence list**

**Table S6: The results of LASSO-COX regression model**

| coef | se | z | p | HR | HRse | HRz | HRp | gene | log_rank_p | adj.  pvalue |
| --- | --- | --- | --- | --- | --- | --- | --- | --- | --- | --- |
| 0.726437869 | 0.110299805 | 6.58603041 | 4.52E-11 | 2.067702047 | 0.228067132 | 4.681525291 | 2.85E-06 | BIRC5 | 1.22E-14 | 2.51E-08 |
| 0.712251359 | 0.110245885 | 6.46057095 | 1.04E-10 | 2.038575662 | 0.224744577 | 4.621137807 | 3.82E-06 | SPHK1 | 3.77E-13 | 2.89E-08 |
| -0.698640561 | 0.110334322 | -6.332032947 | 2.42E-10 | 0.49726084 | 0.054864938 | -9.163213918 | 0 | SPDEF | 9.53E-13 | 4.48E-08 |
| 0.677108544 | 0.110136892 | 6.147881327 | 7.85E-10 | 1.968178594 | 0.216769074 | 4.466405548 | 7.95E-06 | CCNE1 | 3.10E-13 | 9.64E-08 |
| 0.674052338 | 0.109924948 | 6.131932299 | 8.68E-10 | 1.962172618 | 0.215691723 | 4.460869459 | 8.16E-06 | CDC25A | 5.70E-14 | 9.64E-08 |
| 0.671574817 | 0.110073719 | 6.101136822 | 1.05E-09 | 1.957317312 | 0.215449195 | 4.443355254 | 8.86E-06 | CDC20 | 8.13E-14 | 9.74E-08 |
| 0.666542875 | 0.11005856 | 6.056256566 | 1.39E-09 | 1.947492942 | 0.214338268 | 4.420549583 | 9.85E-06 | SRF | 4.60E-12 | 1.03E-07 |
| 0.665483282 | 0.110080977 | 6.045397686 | 1.49E-09 | 1.945430486 | 0.214154888 | 4.414704209 | 1.01E-05 | AURKA | 4.34E-14 | 1.03E-07 |
| 0.66175523 | 0.10982153 | 6.025733143 | 1.68E-09 | 1.938191323 | 0.212855136 | 4.407651806 | 1.04E-05 | MCM2 | 1.82E-13 | 1.04E-07 |
| 0.647416114 | 0.109868544 | 5.892643083 | 3.80E-09 | 1.910597679 | 0.209914584 | 4.33794384 | 1.44E-05 | PTTG1 | 1.62E-12 | 2.11E-07 |
| 0.640827765 | 0.109831225 | 5.834659152 | 5.39E-09 | 1.898051369 | 0.208465308 | 4.307917603 | 1.65E-05 | CDKN3 | 9.39E-13 | 2.52E-07 |
| 0.640758688 | 0.109852108 | 5.832921206 | 5.45E-09 | 1.897920262 | 0.208490541 | 4.306767389 | 1.66E-05 | MYBL2 | 1.92E-12 | 2.52E-07 |
| 0.62556128 | 0.109840857 | 5.695160218 | 1.23E-08 | 1.869294861 | 0.20532495 | 4.233751727 | 2.30E-05 | CIP2A | 5.48E-12 | 5.26E-07 |
| 0.619737896 | 0.109883028 | 5.639978324 | 1.70E-08 | 1.858440873 | 0.20421111 | 4.203693283 | 2.63E-05 | E2F1 | 1.30E-12 | 6.33E-07 |
| 0.619344181 | 0.10983351 | 5.638936454 | 1.71E-08 | 1.857709322 | 0.204038735 | 4.203659286 | 2.63E-05 | FAM83D | 1.31E-11 | 6.33E-07 |
| 0.595381104 | 0.109694355 | 5.427636655 | 5.71E-08 | 1.81372203 | 0.198955069 | 4.089978881 | 4.31E-05 | EZH2 | 2.77E-10 | 1.98E-06 |
| 0.589065329 | 0.109783024 | 5.365723303 | 8.06E-08 | 1.802303067 | 0.19786228 | 4.054856062 | 5.02E-05 | UBE2C | 1.84E-11 | 2.63E-06 |
| 0.584887254 | 0.10962183 | 5.335499834 | 9.53E-08 | 1.794788618 | 0.196748012 | 4.039627186 | 5.35E-05 | CCNA2 | 1.31E-10 | 2.94E-06 |
| 0.574446151 | 0.109599902 | 5.241301702 | 1.59E-07 | 1.776146538 | 0.194665486 | 3.987078316 | 6.69E-05 | CHEK1 | 1.81E-10 | 4.66E-06 |
| 0.567728751 | 0.109723108 | 5.174194966 | 2.29E-07 | 1.764255433 | 0.193579589 | 3.948016621 | 7.88E-05 | CDKN1B | 1.34E-09 | 6.13E-06 |
| 0.566828138 | 0.10960449 | 5.171577723 | 2.32E-07 | 1.762667238 | 0.193196243 | 3.94762975 | 7.89E-05 | LMNB1 | 1.50E-09 | 6.13E-06 |
| 0.557102983 | 0.109642739 | 5.08107502 | 3.75E-07 | 1.745608111 | 0.191393255 | 3.895686462 | 9.79E-05 | MYC | 6.43E-09 | 9.07E-06 |
| 0.556644417 | 0.109558743 | 5.08078499 | 3.76E-07 | 1.744807819 | 0.191158951 | 3.89627487 | 9.77E-05 | AKR1B1 | 7.30E-10 | 9.07E-06 |
| 0.555821412 | 0.109667396 | 5.068246654 | 4.01E-07 | 1.743372424 | 0.191191114 | 3.888111793 | 0.000101027 | GMNN | 2.16E-09 | 9.28E-06 |
| 0.551552695 | 0.109489953 | 5.037473124 | 4.72E-07 | 1.735946322 | 0.190068681 | 3.872002045 | 0.000107945 | YY1 | 3.37E-10 | 1.05E-05 |
| 0.547231079 | 0.109578665 | 4.993956436 | 5.92E-07 | 1.728460416 | 0.189402385 | 3.84609949 | 0.000120013 | TOP2A | 2.12E-09 | 1.26E-05 |
| 0.544696161 | 0.109463123 | 4.976069974 | 6.49E-07 | 1.724084459 | 0.18872367 | 3.836744272 | 0.000124676 | MKI67 | 6.25E-10 | 1.33E-05 |
| 0.542812668 | 0.109579329 | 4.9536046 | 7.29E-07 | 1.720840213 | 0.188568516 | 3.822696544 | 0.000132 | HSPD1 | 2.49E-09 | 1.44E-05 |
| 0.537172634 | 0.109490435 | 4.906114708 | 9.29E-07 | 1.711161935 | 0.187355865 | 3.795781551 | 0.000147179 | PLK1 | 1.02E-09 | 1.78E-05 |
| 0.533098886 | 0.109474461 | 4.869618748 | 1.12E-06 | 1.704205273 | 0.186566953 | 3.774544531 | 0.0001603 | AURKB | 4.10E-09 | 2.02E-05 |
| 0.532876076 | 0.109478927 | 4.867384878 | 1.13E-06 | 1.7038256 | 0.186532999 | 3.77319618 | 0.000161169 | CDK4 | 9.79E-09 | 2.02E-05 |
| 0.530139779 | 0.109512556 | 4.840904069 | 1.29E-06 | 1.6991698 | 0.186080428 | 3.757352705 | 0.00017172 | HMGB1 | 1.40E-08 | 2.24E-05 |
| 0.528811917 | 0.109446794 | 4.831680268 | 1.35E-06 | 1.696915035 | 0.185721911 | 3.752465345 | 0.000175104 | FOXM1 | 2.87E-09 | 2.28E-05 |
| 0.52756967 | 0.109576623 | 4.814618822 | 1.47E-06 | 1.694808355 | 0.185711376 | 3.741334386 | 0.000183046 | FOXK1 | 3.47E-08 | 2.41E-05 |
| 0.522360526 | 0.109574918 | 4.76715415 | 1.87E-06 | 1.686002809 | 0.18474362 | 3.713269286 | 0.000204599 | PSMC4 | 9.07E-09 | 2.96E-05 |
| 0.515947651 | 0.109543088 | 4.709997324 | 2.48E-06 | 1.675225279 | 0.18350935 | 3.679514307 | 0.000233679 | MECP2 | 3.04E-08 | 3.82E-05 |
| 0.507065001 | 0.109930848 | 4.612581548 | 3.98E-06 | 1.660410732 | 0.18253036 | 3.618087058 | 0.000296789 | CALCA | 7.32E-07 | 5.97E-05 |
| 0.494772441 | 0.10936435 | 4.524074255 | 6.07E-06 | 1.640124971 | 0.179371201 | 3.568716525 | 0.000358734 | VDAC1 | 1.42E-07 | 8.86E-05 |
| 0.489985144 | 0.109459394 | 4.476410166 | 7.59E-06 | 1.632291971 | 0.17866969 | 3.538887713 | 0.000401817 | PARD3 | 1.22E-07 | 0.000108024 |
| 0.484451909 | 0.109383486 | 4.428930965 | 9.47E-06 | 1.623285057 | 0.177560579 | 3.510267098 | 0.000447657 | TEAD4 | 1.30E-07 | 0.000131398 |
| 0.482047752 | 0.109411256 | 4.405833267 | 1.05E-05 | 1.619387113 | 0.177179178 | 3.495823375 | 0.000472601 | MAPK14 | 7.33E-08 | 0.000139865 |
| 0.481827553 | 0.109451034 | 4.402220188 | 1.07E-05 | 1.619030564 | 0.17720457 | 3.493310385 | 0.000477072 | PIMREG | 9.79E-09 | 0.000139865 |
| 0.481804069 | 0.109506541 | 4.399774354 | 1.08E-05 | 1.618992543 | 0.177290272 | 3.49140725 | 0.000480483 | GLO1 | 2.64E-08 | 0.000139865 |
| 0.480273319 | 0.109411636 | 4.389600024 | 1.14E-05 | 1.616516167 | 0.176865678 | 3.485787481 | 0.000490691 | TYMS | 2.94E-08 | 0.00014324 |
| 0.477924606 | 0.109286096 | 4.373151064 | 1.22E-05 | 1.612723889 | 0.176248297 | 3.476481181 | 0.00050804 | STMN1 | 5.17E-08 | 0.000151041 |
| 0.475673584 | 0.109289633 | 4.352412658 | 1.35E-05 | 1.609097695 | 0.175857697 | 3.463582797 | 0.000533033 | CSNK2A1 | 2.31E-08 | 0.000162455 |
| 0.474747575 | 0.109331808 | 4.342264025 | 1.41E-05 | 1.607608346 | 0.175762727 | 3.456980659 | 0.000546264 | CDC6 | 9.34E-08 | 0.000166526 |
| 0.472565775 | 0.109382734 | 4.320295889 | 1.56E-05 | 1.60410469 | 0.175461356 | 3.442950075 | 0.000575406 | PRDX1 | 1.41E-07 | 0.000180167 |
| 0.471491699 | 0.109391911 | 4.310114858 | 1.63E-05 | 1.602382684 | 0.175287703 | 3.436537034 | 0.000589202 | MTDH | 3.37E-07 | 0.000184815 |
| 0.469770108 | 0.109306326 | 4.297739416 | 1.73E-05 | 1.599626409 | 0.174849286 | 3.429390083 | 0.000604939 | NEK2 | 2.77E-07 | 0.000191529 |
| 0.468198845 | 0.109327837 | 4.282521784 | 1.85E-05 | 1.597114949 | 0.174609123 | 3.419723653 | 0.000626848 | DNMT3B | 2.61E-07 | 0.000201092 |
| 0.465454324 | 0.109298201 | 4.2585726 | 2.06E-05 | 1.592737643 | 0.174083359 | 3.404906974 | 0.000661866 | RHOA | 3.21E-07 | 0.000219584 |
| 0.462552948 | 0.109354466 | 4.229849632 | 2.34E-05 | 1.588123209 | 0.173668365 | 3.386472878 | 0.000707973 | TUBB | 3.82E-07 | 0.000244878 |
| 0.457426259 | 0.109654921 | 4.171506911 | 3.03E-05 | 1.580002231 | 0.173255019 | 3.347679241 | 0.000814913 | SNCA | 5.11E-06 | 0.000310997 |
| 0.455095303 | 0.109244682 | 4.16583483 | 3.10E-05 | 1.576323605 | 0.172204972 | 3.346730347 | 0.000817707 | PSMC1 | 1.40E-07 | 0.000313035 |
| 0.45392729 | 0.109234795 | 4.155519217 | 3.25E-05 | 1.574483513 | 0.171988384 | 3.340246009 | 0.000837042 | MCM5 | 2.02E-07 | 0.000316753 |
| 0.454270424 | 0.109331524 | 4.154981161 | 3.25E-05 | 1.575023864 | 0.17219976 | 3.339283773 | 0.000839947 | SKP1 | 8.95E-07 | 0.000316753 |
| 0.45179751 | 0.109261326 | 4.135017624 | 3.55E-05 | 1.571133778 | 0.171664161 | 3.327041452 | 0.000877733 | PAX8 | 5.02E-07 | 0.000339629 |
| 0.45104459 | 0.109210547 | 4.130046085 | 3.63E-05 | 1.569951285 | 0.171455238 | 3.324198736 | 0.00088673 | HSPA4 | 2.49E-07 | 0.000341175 |
| 0.451022571 | 0.109352162 | 4.124496139 | 3.72E-05 | 1.569916716 | 0.171673787 | 3.319765497 | 0.000900931 | SIRT2 | 5.55E-07 | 0.000343681 |
| 0.4472237 | 0.109232074 | 4.094252571 | 4.24E-05 | 1.563964119 | 0.170835044 | 3.301220313 | 0.000962653 | KDM1A | 5.09E-07 | 0.000385345 |
| 0.441500227 | 0.109334187 | 4.038080294 | 5.39E-05 | 1.55503838 | 0.170018857 | 3.264569522 | 0.001096306 | USP11 | 1.42E-06 | 0.000482406 |
| 0.440974513 | 0.10939555 | 4.031009589 | 5.55E-05 | 1.554221089 | 0.170024871 | 3.259647157 | 0.001115509 | PIK3CA | 5.16E-07 | 0.000489261 |
| 0.436715906 | 0.109414415 | 3.991392764 | 6.57E-05 | 1.547616346 | 0.169331538 | 3.233989092 | 0.001220741 | POSTN | 2.71E-05 | 0.000569624 |
| 0.436313028 | 0.109691847 | 3.977624933 | 6.96E-05 | 1.54699297 | 0.169692517 | 3.223436013 | 0.001266626 | NTS | 5.17E-06 | 0.000594337 |
| 0.432208855 | 0.109285195 | 3.954871063 | 7.66E-05 | 1.540656855 | 0.168370985 | 3.21110467 | 0.001322258 | CRKL | 1.45E-06 | 0.000643934 |
| 0.432778251 | 0.109617584 | 3.948073234 | 7.88E-05 | 1.541534349 | 0.168979271 | 3.204738332 | 0.001351853 | FAP | 7.39E-05 | 0.000652603 |
| 0.43135694 | 0.109455443 | 3.940936416 | 8.12E-05 | 1.539344905 | 0.168489678 | 3.201056064 | 0.001369249 | KLF8 | 1.18E-06 | 0.000662443 |
| 0.428711495 | 0.109170512 | 3.926989885 | 8.60E-05 | 1.535278035 | 0.167607088 | 3.193647951 | 0.001404873 | PRMT5 | 1.43E-06 | 0.00068096 |
| 0.429416142 | 0.109400347 | 3.925180814 | 8.67E-05 | 1.536360246 | 0.168078344 | 3.191132385 | 0.001417163 | IKBKB | 2.05E-06 | 0.00068096 |
| 0.428509102 | 0.10920389 | 3.923936266 | 8.71E-05 | 1.534967338 | 0.167624404 | 3.191464527 | 0.001415535 | E2F3 | 3.90E-07 | 0.00068096 |
| 0.426920074 | 0.109363025 | 3.903696635 | 9.47E-05 | 1.532530167 | 0.167602135 | 3.177347151 | 0.00148629 | MCL1 | 2.59E-06 | 0.000730245 |
| 0.421026508 | 0.109236148 | 3.854278229 | 0.000116072 | 1.523524664 | 0.166423966 | 3.145728804 | 0.001656736 | NRAS | 1.95E-06 | 0.000882462 |
| 0.415996981 | 0.109178644 | 3.810241336 | 0.000138831 | 1.515881292 | 0.165501863 | 3.117072411 | 0.001826567 | MTA2 | 5.07E-06 | 0.001041234 |
| 0.414935271 | 0.109166277 | 3.800947349 | 0.000144144 | 1.51427272 | 0.165307515 | 3.111006295 | 0.00186451 | PCNA | 1.50E-06 | 0.001066665 |
| 0.414550228 | 0.109201723 | 3.796187597 | 0.000146938 | 1.513689772 | 0.165297532 | 3.107667529 | 0.001885701 | FERMT2 | 2.97E-06 | 0.001073036 |
| 0.41116525 | 0.109227761 | 3.764292576 | 0.000167021 | 1.508574628 | 0.164778229 | 3.086418825 | 0.002025833 | CEBPA | 8.18E-07 | 0.001185968 |
| 0.41098037 | 0.109203396 | 3.763439456 | 0.000167592 | 1.508295748 | 0.164711018 | 3.085985103 | 0.00202879 | HMGA2 | 8.18E-06 | 0.001185968 |
| -0.410953264 | 0.109248884 | -3.761624359 | 0.000168813 | 0.663017917 | 0.072433968 | -4.652265961 | 3.28E-06 | TFF1 | 4.39E-06 | 0.001185968 |
| 0.409514823 | 0.109319027 | 3.746052581 | 0.000179639 | 1.50608689 | 0.164643953 | 3.073826152 | 0.002113325 | SNAI1 | 6.10E-06 | 0.001246245 |
| -0.409280706 | 0.109535026 | -3.736528117 | 0.000186579 | 0.664127782 | 0.072745254 | -4.617101453 | 3.89E-06 | PGR | 6.71E-06 | 0.001278409 |
| -0.406151218 | 0.109232491 | -3.718227194 | 0.000200626 | 0.666209417 | 0.072771714 | -4.586817658 | 4.50E-06 | MSX1 | 2.83E-06 | 0.001357894 |
| 0.404941144 | 0.109209199 | 3.707939877 | 0.000208952 | 1.499214259 | 0.163727988 | 3.049046558 | 0.002295689 | FZD7 | 7.55E-06 | 0.001397211 |
| 0.402133586 | 0.109171621 | 3.683499264 | 0.000230054 | 1.495011031 | 0.163212778 | 3.032918368 | 0.002422011 | PKM | 1.29E-05 | 0.0015062 |
| -0.402370824 | 0.109256542 | -3.682807626 | 0.000230679 | 0.668732717 | 0.073063424 | -4.533968776 | 5.79E-06 | NT5E | 1.23E-05 | 0.0015062 |
| 0.399939047 | 0.109174749 | 3.663292571 | 0.000248994 | 1.49173377 | 0.16285966 | 3.019371216 | 0.002533 | FLNA | 8.12E-06 | 0.001606879 |
| 0.397900951 | 0.109191634 | 3.644060793 | 0.00026837 | 1.488696569 | 0.162553211 | 3.006379066 | 0.002643792 | TIMP2 | 4.45E-06 | 0.001712015 |
| -0.397401624 | 0.109347006 | -3.634316481 | 0.000278719 | 0.672064054 | 0.073488192 | -4.462430462 | 8.10E-06 | FBP1 | 4.03E-05 | 0.001744083 |
| 0.396441243 | 0.109109463 | 3.633426773 | 0.000279682 | 1.486525091 | 0.162193954 | 2.999649984 | 0.0027029 | SMAD2 | 1.12E-05 | 0.001744083 |
| 0.396293054 | 0.109156281 | 3.630510784 | 0.000282861 | 1.486304821 | 0.162239506 | 2.997450083 | 0.002722484 | NME1 | 2.34E-05 | 0.001744309 |
| 0.395619173 | 0.109206277 | 3.622677958 | 0.000291569 | 1.485303567 | 0.162204473 | 2.991924684 | 0.002772246 | CUL1 | 3.72E-05 | 0.001778249 |
| 0.394859038 | 0.109243428 | 3.614487808 | 0.000300942 | 1.484174964 | 0.162136361 | 2.986220748 | 0.002824487 | SP1 | 3.84E-05 | 0.001815465 |
| 0.393390347 | 0.109138742 | 3.604497709 | 0.000312757 | 1.481996769 | 0.161743264 | 2.980011399 | 0.002882377 | PTK2 | 4.77E-06 | 0.001866454 |
| 0.390631533 | 0.109247439 | 3.5756585 | 0.000349347 | 1.477913851 | 0.161458303 | 2.959983117 | 0.003076559 | ILK | 0.000106885 | 0.002026794 |
| 0.390365141 | 0.109251219 | 3.573096436 | 0.000352785 | 1.477520198 | 0.161420882 | 2.958230625 | 0.003094105 | RPS27A | 5.41E-05 | 0.002026794 |
| 0.390056565 | 0.109176249 | 3.572723634 | 0.000353288 | 1.477064342 | 0.161260344 | 2.958348772 | 0.00309292 | NAMPT | 2.68E-05 | 0.002026794 |
| 0.389786488 | 0.109122013 | 3.572024347 | 0.000354232 | 1.476665474 | 0.161136709 | 2.958143282 | 0.003094982 | GAPDH | 2.13E-05 | 0.002026794 |
| 0.38964844 | 0.109200097 | 3.568205976 | 0.000359434 | 1.476461638 | 0.161229755 | 2.955171885 | 0.003124949 | ENO1 | 1.29E-05 | 0.00203557 |
| 0.386351854 | 0.10915668 | 3.539424744 | 0.000401 | 1.47160237 | 0.160635229 | 2.935858917 | 0.003326256 | CUL3 | 3.81E-05 | 0.002248031 |
| 0.384833503 | 0.109246105 | 3.52262905 | 0.000427289 | 1.469369655 | 0.160522912 | 2.924004123 | 0.003455602 | AKT2 | 1.15E-05 | 0.002371454 |
| 0.383549855 | 0.109241132 | 3.511038802 | 0.000446359 | 1.467484712 | 0.160309692 | 2.916135055 | 0.003543971 | RAF1 | 1.11E-05 | 0.002447652 |
| 0.383067138 | 0.109167843 | 3.508974131 | 0.000449839 | 1.466776503 | 0.160124827 | 2.915078884 | 0.003555986 | TGFBR1 | 3.69E-05 | 0.002447652 |
| 0.381076352 | 0.109199555 | 3.489724414 | 0.000483519 | 1.463859369 | 0.159852791 | 2.901790867 | 0.003710361 | GSK3B | 8.90E-06 | 0.002580605 |
| 0.380823326 | 0.109127981 | 3.489694593 | 0.000483573 | 1.463489022 | 0.159707603 | 2.902109949 | 0.003706584 | PPP2CA | 2.75E-05 | 0.002580605 |
| 0.379442233 | 0.109117329 | 3.477378299 | 0.000506343 | 1.461469203 | 0.159471616 | 2.893738795 | 0.003806847 | UHRF1 | 4.92E-05 | 0.002676384 |
| 0.378599632 | 0.109141134 | 3.468899561 | 0.000522595 | 1.460238286 | 0.159372062 | 2.887822864 | 0.003879183 | BRCA1 | 1.03E-05 | 0.002736227 |
| 0.378266168 | 0.109162846 | 3.465154873 | 0.000529926 | 1.459751431 | 0.159350621 | 2.885156194 | 0.003912195 | HNRNPK | 7.31E-05 | 0.002748683 |
| 0.37747794 | 0.109115244 | 3.459442749 | 0.000541294 | 1.458601267 | 0.159155633 | 2.881464246 | 0.003958321 | EIF4E | 4.60E-05 | 0.002759157 |
| 0.377636606 | 0.109170433 | 3.459147286 | 0.000541888 | 1.458832716 | 0.159261399 | 2.881003928 | 0.003964107 | PAWR | 9.11E-05 | 0.002759157 |
| 0.376639305 | 0.109161018 | 3.450309577 | 0.000559944 | 1.457378545 | 0.159088925 | 2.874986707 | 0.004040445 | MMP1 | 7.75E-05 | 0.002810976 |
| 0.376308225 | 0.109099313 | 3.449226363 | 0.000562195 | 1.456896116 | 0.158946365 | 2.874530142 | 0.004046291 | CTNNAL1 | 2.17E-05 | 0.002810976 |
| 0.375941213 | 0.109299914 | 3.439538052 | 0.000582708 | 1.456361516 | 0.159180188 | 2.866949216 | 0.004144495 | CDKN2A | 8.45E-05 | 0.002887526 |
| 0.374082333 | 0.109220401 | 3.425022521 | 0.000614748 | 1.45365683 | 0.158768982 | 2.857339162 | 0.004272091 | GPI | 1.13E-05 | 0.00301934 |
| 0.372362816 | 0.109173029 | 3.410758302 | 0.000647825 | 1.451159389 | 0.158427466 | 2.847734674 | 0.004403162 | ZFYVE9 | 4.80E-05 | 0.003130383 |
| 0.372271833 | 0.109157301 | 3.410416257 | 0.000648638 | 1.451027365 | 0.15839023 | 2.847570608 | 0.004405432 | TRIM33 | 3.21E-05 | 0.003130383 |
| 0.369756789 | 0.109201181 | 3.386014559 | 0.000709156 | 1.447382552 | 0.158055884 | 2.83053399 | 0.004647037 | CREB1 | 4.46E-05 | 0.003392946 |
| 0.368984487 | 0.109101865 | 3.382018158 | 0.000719554 | 1.446265168 | 0.157790227 | 2.828218057 | 0.004680791 | TFRC | 1.59E-05 | 0.003413268 |
| 0.365809328 | 0.109128092 | 3.352109646 | 0.000801982 | 1.441680329 | 0.157327823 | 2.807388551 | 0.004994496 | APC | 7.44E-05 | 0.003772036 |
| -0.36462923 | 0.109353158 | -3.334418848 | 0.000854778 | 0.694454086 | 0.075940747 | -4.02347785 | 5.73E-05 | AQP5 | 0.00038633 | 0.003943219 |
| 0.363817693 | 0.109132916 | 3.333711837 | 0.000856954 | 1.438811884 | 0.157021736 | 2.794593255 | 0.005196505 | NOTCH2 | 8.61E-05 | 0.003943219 |
| 0.363772538 | 0.109148437 | 3.332824082 | 0.000859693 | 1.438746917 | 0.157036977 | 2.793908327 | 0.005207524 | SRC | 7.60E-05 | 0.003943219 |
| 0.362325359 | 0.109159721 | 3.319222113 | 0.000902686 | 1.436666299 | 0.156826092 | 2.784398264 | 0.005362714 | CUL7 | 4.13E-05 | 0.004106481 |
| -0.360804066 | 0.109240789 | -3.302832834 | 0.000957134 | 0.697115574 | 0.076153456 | -3.977290642 | 6.97E-05 | ESR1 | 8.46E-05 | 0.004227079 |
| 0.361142057 | 0.109347168 | 3.302710655 | 0.000957551 | 1.434967294 | 0.15690961 | 2.772088301 | 0.005569793 | ALK | 2.10E-05 | 0.004227079 |
| 0.360348813 | 0.109113119 | 3.302525077 | 0.000958185 | 1.433829465 | 0.156449606 | 2.772966182 | 0.005554789 | CTNNB1 | 0.000127871 | 0.004227079 |
| 0.36017036 | 0.109073341 | 3.302093386 | 0.000959661 | 1.433573617 | 0.156364665 | 2.77283629 | 0.005557007 | CUL4A | 6.26E-05 | 0.004227079 |
| 0.359422644 | 0.109161669 | 3.292571899 | 0.000992755 | 1.432502112 | 0.156374321 | 2.765812878 | 0.005678111 | SKP2 | 3.24E-05 | 0.004338418 |
| 0.359319902 | 0.109223184 | 3.289776848 | 0.001002669 | 1.432354942 | 0.156446367 | 2.763598471 | 0.005716784 | RAC1 | 0.000211873 | 0.004347509 |
| 0.358201999 | 0.109100498 | 3.283229719 | 0.00102625 | 1.430754602 | 0.15609604 | 2.759548554 | 0.005788129 | KLF6 | 9.65E-05 | 0.004415262 |
| 0.357556062 | 0.109094233 | 3.277497371 | 0.001047317 | 1.429830723 | 0.155986286 | 2.75556739 | 0.005859043 | PARP1 | 4.11E-05 | 0.004471238 |
| 0.35591513 | 0.109043257 | 3.263981102 | 0.001098585 | 1.427486392 | 0.155657766 | 2.746322299 | 0.006026753 | RASSF1 | 9.84E-05 | 0.00465431 |
| 0.352852689 | 0.10911259 | 3.233840287 | 0.001221377 | 1.423121487 | 0.155280471 | 2.724885386 | 0.006432381 | ITGAV | 0.000135136 | 0.005131582 |
| 0.352770326 | 0.109152844 | 3.231893131 | 0.00122973 | 1.423004279 | 0.155324964 | 2.72335025 | 0.006462351 | CLDN1 | 5.06E-05 | 0.005131582 |
| 0.351662167 | 0.109147685 | 3.221893046 | 0.001273467 | 1.421428237 | 0.155145601 | 2.716340222 | 0.006600805 | ROCK1 | 4.68E-05 | 0.005274433 |
| 0.349818857 | 0.109082214 | 3.20692845 | 0.001341604 | 1.418810517 | 0.154766993 | 2.706071296 | 0.006808443 | SHC1 | 4.55E-05 | 0.005515481 |
| 0.34744451 | 0.109095428 | 3.184776091 | 0.00144866 | 1.415445766 | 0.154418662 | 2.690385747 | 0.007136947 | BMP2 | 0.000129027 | 0.005911813 |
| 0.345151858 | 0.109128001 | 3.162816633 | 0.001562507 | 1.412204358 | 0.154111039 | 2.67472311 | 0.007479099 | MAP3K7 | 6.95E-05 | 0.006283996 |
| 0.345151858 | 0.109128001 | 3.162816633 | 0.001562507 | 1.412204358 | 0.154111039 | 2.67472311 | 0.007479099 | ESRRA | 6.95E-05 | 0.006283996 |
| -0.343354958 | 0.109130131 | -3.146289247 | 0.001653564 | 0.709386365 | 0.077415427 | -3.753949916 | 0.00017407 | ERG | 0.000422779 | 0.006602359 |
| 0.342877957 | 0.109112435 | 3.142427862 | 0.00167553 | 1.408996793 | 0.153739072 | 2.660330839 | 0.007806393 | WWTR1 | 0.000234858 | 0.006642279 |
| 0.342134513 | 0.109078944 | 3.136577043 | 0.001709325 | 1.407949672 | 0.153577664 | 2.656308616 | 0.007900128 | PTPN11 | 7.46E-05 | 0.006728195 |
| -0.339815525 | 0.109308199 | -3.108783505 | 0.001878593 | 0.711901639 | 0.077816686 | -3.702269745 | 0.000213679 | FOXA2 | 0.000130546 | 0.007342389 |
| 0.338528893 | 0.109062374 | 3.103993432 | 0.001909276 | 1.402882282 | 0.153001672 | 2.633188747 | 0.008458733 | CDC42 | 0.000258613 | 0.007410126 |
| 0.338411079 | 0.109112294 | 3.101493565 | 0.001925471 | 1.402717013 | 0.153053672 | 2.631214319 | 0.008508036 | YAP1 | 0.000263659 | 0.007421084 |
| 0.338041131 | 0.109112256 | 3.098104139 | 0.00194763 | 1.402198176 | 0.152997006 | 2.628797692 | 0.008568732 | ITGB1 | 0.000631873 | 0.007454721 |
| 0.336582672 | 0.109043 | 3.086696719 | 0.00202394 | 1.400154618 | 0.152677061 | 2.620921676 | 0.008769241 | SUZ12 | 0.000129004 | 0.007693744 |
| 0.335930145 | 0.109138774 | 3.078009143 | 0.002083885 | 1.399241277 | 0.152711478 | 2.614350165 | 0.008939736 | SMURF2 | 0.000556343 | 0.00786773 |
| 0.334389328 | 0.109168675 | 3.063051996 | 0.00219092 | 1.397086963 | 0.152518133 | 2.603539369 | 0.009226667 | PRNP | 8.28E-05 | 0.00821595 |
| 0.334017488 | 0.109194893 | 3.058911254 | 0.002221429 | 1.396567567 | 0.152498047 | 2.600476372 | 0.009309443 | TGFB1 | 0.0007947 | 0.008274452 |
| 0.33326668 | 0.109048684 | 3.056127468 | 0.002242159 | 1.395519405 | 0.152179555 | 2.599031158 | 0.009348729 | PAK4 | 5.57E-05 | 0.008295989 |
| 0.332962165 | 0.109161476 | 3.050180119 | 0.002287042 | 1.395094514 | 0.152290577 | 2.594346428 | 0.009477094 | CHRNA7 | 0.000735872 | 0.008364279 |
| 0.333056571 | 0.109209885 | 3.04969253 | 0.002290758 | 1.395226225 | 0.152372496 | 2.593816052 | 0.009491726 | FGFR4 | 0.000988623 | 0.008364279 |
| 0.328923683 | 0.109111552 | 3.014563332 | 0.002573495 | 1.389471811 | 0.151607425 | 2.568949444 | 0.010200734 | CCND3 | 2.34E-05 | 0.009294763 |
| 0.328661192 | 0.1090483 | 3.013904786 | 0.002579087 | 1.389107136 | 0.151479771 | 2.568706914 | 0.010207875 | ESRP1 | 0.000148561 | 0.009294763 |
| 0.327701414 | 0.109187179 | 3.001281083 | 0.002688463 | 1.387774541 | 0.151527187 | 2.559108689 | 0.010494093 | SMAD3 | 0.000961792 | 0.009626431 |
| 0.327036427 | 0.109066876 | 2.998494498 | 0.002713171 | 1.386851995 | 0.151259614 | 2.55753657 | 0.010541647 | SMAD4 | 0.000726177 | 0.009652626 |
| 0.326638535 | 0.109069871 | 2.994764112 | 0.002746572 | 1.386300288 | 0.151203593 | 2.554835365 | 0.010623803 | CDK6 | 0.000413874 | 0.009709218 |
| 0.325136426 | 0.109010348 | 2.982619844 | 0.002857927 | 1.384219477 | 0.150894246 | 2.546283149 | 0.010887683 | PSMD10 | 0.000138505 | 0.010038922 |
| 0.324922072 | 0.109090111 | 2.97847412 | 0.002896875 | 1.383922795 | 0.150972291 | 2.543001715 | 0.010990469 | PRKCZ | 0.000729225 | 0.010111733 |
| 0.323918314 | 0.10907567 | 2.969666042 | 0.002981237 | 1.382534368 | 0.150800863 | 2.536685542 | 0.011190742 | PLK4 | 0.000302828 | 0.010341164 |
| 0.323339846 | 0.109078323 | 2.964290583 | 0.003033817 | 1.381734849 | 0.150717321 | 2.532786859 | 0.011315974 | MAP2K1 | 0.000283112 | 0.010457309 |
| 0.323144213 | 0.109081505 | 2.962410652 | 0.003052404 | 1.381464562 | 0.150692234 | 2.531414872 | 0.011360339 | MAD2L2 | 0.000430571 | 0.010457309 |
| 0.322829435 | 0.109161828 | 2.957347272 | 0.003102984 | 1.381029776 | 0.150755735 | 2.527464544 | 0.011488942 | EFEMP1 | 0.001257031 | 0.010565376 |
| 0.321344028 | 0.108961669 | 2.949147455 | 0.003186519 | 1.378979906 | 0.150255952 | 2.52222892 | 0.011661379 | JUP | 0.000161333 | 0.010783647 |
| 0.320632205 | 0.109141196 | 2.937774352 | 0.003305775 | 1.377998667 | 0.150396422 | 2.513348801 | 0.011959103 | LATS2 | 0.000243429 | 0.011115155 |
| 0.321613732 | 0.109550153 | 2.935767066 | 0.003327241 | 1.379351874 | 0.151108209 | 2.510465021 | 0.012057227 | STAT1 | 9.19E-05 | 0.011115155 |
| 0.31973189 | 0.108968946 | 2.934156044 | 0.00334456 | 1.376758592 | 0.150023932 | 2.511323274 | 0.012027949 | CLDN7 | 0.000185762 | 0.011115155 |
| 0.319979798 | 0.109152179 | 2.931501701 | 0.003373275 | 1.377099944 | 0.15031346 | 2.508757002 | 0.01211568 | SYP | 0.000279037 | 0.011143856 |
| 0.318438732 | 0.109199287 | 2.916124649 | 0.003544089 | 1.374979376 | 0.150146767 | 2.497418913 | 0.012510107 | NDRG1 | 0.000123635 | 0.011576588 |
| 0.318189141 | 0.109119931 | 2.915958046 | 0.003545982 | 1.374636236 | 0.150000211 | 2.497571392 | 0.012504728 | ITGA5 | 0.000237817 | 0.011576588 |
| 0.316257796 | 0.109130155 | 2.897987226 | 0.003755659 | 1.371983902 | 0.149724816 | 2.484450549 | 0.012975153 | PRKD1 | 0.001590599 | 0.012189419 |
| 0.315263644 | 0.108988352 | 2.892636129 | 0.003820236 | 1.37062062 | 0.149381682 | 2.481031233 | 0.013100289 | SLC25A4 | 0.000262452 | 0.012326924 |
| 0.314974444 | 0.109039433 | 2.888628777 | 0.003869256 | 1.370224293 | 0.14940848 | 2.477933602 | 0.013214573 | DDR1 | 0.000670396 | 0.01241293 |
| 0.313410576 | 0.109205953 | 2.869903753 | 0.004105968 | 1.368083117 | 0.149402821 | 2.463695895 | 0.013751273 | ACTC1 | 0.002513053 | 0.013096621 |
| 0.311667413 | 0.109135925 | 2.855772863 | 0.004293221 | 1.365700403 | 0.149046977 | 2.453591554 | 0.014143753 | SMURF1 | 0.000203082 | 0.013615645 |
| 0.310935464 | 0.109008339 | 2.8524007 | 0.004339037 | 1.364701145 | 0.148763806 | 2.451544876 | 0.014224445 | SMARCA4 | 0.000627716 | 0.013682758 |
| 0.309175084 | 0.109051298 | 2.83513437 | 0.004580641 | 1.362300867 | 0.148560678 | 2.438739988 | 0.014738569 | SKI | 0.00038633 | 0.014363028 |
| 0.30903351 | 0.109232168 | 2.829143791 | 0.004667272 | 1.362108014 | 0.148786012 | 2.433750393 | 0.014943296 | WT1 | 0.002517491 | 0.014494104 |
| -0.30859794 | 0.109097769 | -2.828636574 | 0.004674675 | 0.734476014 | 0.080129695 | -3.313677746 | 0.000920776 | PIK3R1 | 0.000663523 | 0.014494104 |
| 0.304833972 | 0.10899306 | 2.796820025 | 0.005160827 | 1.356399784 | 0.147838163 | 2.410742785 | 0.015920072 | HSPA5 | 0.000438405 | 0.015912551 |
| 0.305083375 | 0.109288636 | 2.791537942 | 0.005245821 | 1.356738117 | 0.148276059 | 2.405905037 | 0.016132458 | EPB41L3 | 0.00332583 | 0.016085251 |
| 0.303817395 | 0.10916342 | 2.783142891 | 0.005383509 | 1.3550216 | 0.147918791 | 2.400111551 | 0.016390076 | ERBB2 | 0.000507265 | 0.016416745 |
| 0.302401817 | 0.109072244 | 2.772491012 | 0.005562906 | 1.353104818 | 0.147586179 | 2.392533094 | 0.016732519 | DDR2 | 0.001956292 | 0.016871107 |
| 0.301828294 | 0.109022285 | 2.768500886 | 0.005631483 | 1.352329003 | 0.147433998 | 2.389740541 | 0.016860279 | SQSTM1 | 0.000362211 | 0.016986267 |
| 0.301438998 | 0.109052995 | 2.764151501 | 0.005707103 | 1.351802651 | 0.147418127 | 2.386427352 | 0.017012968 | MSN | 0.000311212 | 0.017069843 |
| 0.301844047 | 0.109230225 | 2.763374753 | 0.005720704 | 1.352350307 | 0.147717529 | 2.385297872 | 0.017065297 | BCL2L1 | 0.000184734 | 0.017069843 |
| 0.300347708 | 0.109042759 | 2.754403047 | 0.005879931 | 1.350328245 | 0.147243517 | 2.379243932 | 0.017348192 | BMI1 | 0.001469437 | 0.017451132 |
| 0.300308246 | 0.109183306 | 2.750495986 | 0.005950512 | 1.35027496 | 0.147427485 | 2.375913561 | 0.017505563 | IL6 | 0.002336651 | 0.017566671 |
| 0.298968591 | 0.109114007 | 2.739965275 | 0.006144568 | 1.348467269 | 0.147136667 | 2.368323783 | 0.01786889 | TERT | 0.004279194 | 0.018043571 |
| 0.297562889 | 0.109059413 | 2.728447546 | 0.006363321 | 1.346573057 | 0.146856468 | 2.359944118 | 0.018277688 | MCAM | 0.001180635 | 0.018516544 |
| 0.297223285 | 0.109013114 | 2.726491113 | 0.006401168 | 1.346115834 | 0.146744278 | 2.358632568 | 0.018342407 | BMPR2 | 0.001073741 | 0.018516544 |
| 0.297658958 | 0.109182308 | 2.726256314 | 0.006405723 | 1.346702427 | 0.14703608 | 2.357941178 | 0.018376605 | KRT7 | 0.000373848 | 0.018516544 |
| 0.296968454 | 0.10909825 | 2.722027655 | 0.006488271 | 1.345772845 | 0.146821462 | 2.355056538 | 0.018519888 | GREM1 | 0.003921123 | 0.018657982 |
| 0.296461314 | 0.108997818 | 2.71988302 | 0.006530501 | 1.345090522 | 0.146611932 | 2.353768337 | 0.018584189 | XIAP | 0.000828709 | 0.01868262 |
| 0.295060568 | 0.108960207 | 2.70796629 | 0.00676969 | 1.343207711 | 0.14635619 | 2.345016705 | 0.019026227 | MAPK7 | 0.001149103 | 0.019267579 |
| 0.294405684 | 0.109001125 | 2.700941693 | 0.006914346 | 1.342328355 | 0.146315301 | 2.339662028 | 0.019301197 | LDHA | 0.000900829 | 0.019578888 |
| 0.294237455 | 0.109297052 | 2.69208959 | 0.007100587 | 1.342102555 | 0.146687852 | 2.332180542 | 0.019691195 | IL10 | 0.001907659 | 0.020004191 |
| 0.292608505 | 0.109038963 | 2.683522453 | 0.007285108 | 1.339918116 | 0.146103282 | 2.326560448 | 0.019988672 | BAX | 0.002777712 | 0.020346565 |
| 0.292508963 | 0.109021113 | 2.68304877 | 0.007295435 | 1.339784745 | 0.146064824 | 2.326259914 | 0.020004689 | WEE1 | 0.002428974 | 0.020346565 |
| -0.292064906 | 0.109107925 | -2.676844115 | 0.00743192 | 0.746720068 | 0.081473077 | -3.108756191 | 0.001878767 | KLK4 | 0.000319211 | 0.020623579 |
| 0.291297649 | 0.109028924 | 2.671746534 | 0.007545762 | 1.338162827 | 0.145898453 | 2.317795844 | 0.020460419 | EZR | 0.000501971 | 0.020835313 |
| 0.290470687 | 0.108991781 | 2.665069642 | 0.007697238 | 1.337056675 | 0.145728188 | 2.312913368 | 0.020727403 | IQGAP1 | 0.001546496 | 0.021148352 |
| 0.289845459 | 0.109031493 | 2.658364578 | 0.00785209 | 1.336220972 | 0.145690168 | 2.307780798 | 0.021011331 | KRT19 | 0.000925614 | 0.021467537 |
| 0.287644975 | 0.108999501 | 2.638956811 | 0.008316157 | 1.333283871 | 0.145327276 | 2.293333223 | 0.021828828 | LATS1 | 0.002103808 | 0.02262484 |
| 0.286807202 | 0.109058629 | 2.629844186 | 0.008542401 | 1.332167349 | 0.145284345 | 2.286325822 | 0.022235204 | DAB2IP | 0.004503216 | 0.023126988 |
| 0.285687591 | 0.108984667 | 2.621355814 | 0.00875808 | 1.330676675 | 0.145023354 | 2.280161536 | 0.02259811 | ADAM10 | 0.002444157 | 0.023595798 |
| -0.284706155 | 0.109020152 | -2.61150027 | 0.009014592 | 0.752235262 | 0.082008802 | -3.021196882 | 0.002517776 | NDRG2 | 0.003731214 | 0.024096595 |
| 0.284558925 | 0.109050754 | 2.609417304 | 0.009069656 | 1.329175633 | 0.144947605 | 2.270997387 | 0.023147136 | FOXO1 | 0.004740876 | 0.024096595 |
| 0.284276897 | 0.108949849 | 2.609245438 | 0.009074213 | 1.328800821 | 0.144772649 | 2.271152894 | 0.023137724 | PCBP1 | 0.000997425 | 0.024096595 |
| 0.284267877 | 0.109075476 | 2.60615757 | 0.009156433 | 1.328788835 | 0.144938275 | 2.268474882 | 0.023300278 | KDM8 | 0.001357382 | 0.024199144 |
| 0.283650436 | 0.109068921 | 2.600653173 | 0.009304647 | 1.327968639 | 0.144840107 | 2.264349611 | 0.023552621 | SMAD7 | 0.004122121 | 0.024474309 |
| 0.281408252 | 0.108998292 | 2.581767547 | 0.009829577 | 1.324994425 | 0.144422129 | 2.250309062 | 0.024429333 | TUBB3 | 0.00236853 | 0.02573309 |
| 0.280713373 | 0.109029837 | 2.574647278 | 0.010034236 | 1.324074034 | 0.144363576 | 2.244846262 | 0.024778004 | SOCS3 | 0.001091215 | 0.026145544 |
| 0.280309761 | 0.109012519 | 2.571353864 | 0.010130176 | 1.32353973 | 0.1442824 | 2.24240608 | 0.024935139 | FN1 | 0.003383392 | 0.026272185 |
| 0.278101333 | 0.108977018 | 2.551926445 | 0.010712913 | 1.320620013 | 0.14391723 | 2.227808387 | 0.025893295 | TSC1 | 0.002516007 | 0.027584646 |
| 0.277931819 | 0.108942125 | 2.551187798 | 0.010735646 | 1.320396168 | 0.143846764 | 2.227343595 | 0.025924319 | TCF3 | 0.001635468 | 0.027584646 |
| 0.277612752 | 0.109012293 | 2.546618765 | 0.010877219 | 1.31997494 | 0.143893495 | 2.223692875 | 0.026169115 | CEP164 | 0.001065246 | 0.027765881 |
| 0.277551484 | 0.109028051 | 2.545688771 | 0.010906238 | 1.319894071 | 0.143905478 | 2.222945759 | 0.026219458 | HOXB9 | 0.003743013 | 0.027765881 |
| -0.276891112 | 0.109018311 | -2.539858763 | 0.011089724 | 0.758137045 | 0.08265082 | -2.926322515 | 0.003429952 | RARA | 0.005335172 | 0.027983873 |
| 0.276909174 | 0.109062062 | 2.539005483 | 0.011116808 | 1.319046562 | 0.143857938 | 2.217789062 | 0.026569218 | DES | 0.006584593 | 0.027983873 |
| 0.276499252 | 0.108936114 | 2.538178048 | 0.011143128 | 1.318505966 | 0.143632916 | 2.217499833 | 0.026588954 | CDK5 | 0.000974529 | 0.027983873 |
| 0.274422252 | 0.108956781 | 2.518633994 | 0.011781105 | 1.315770272 | 0.143362093 | 2.202606454 | 0.027622499 | NFKB1 | 0.002639442 | 0.029452762 |
| 0.273225088 | 0.108996136 | 2.506741046 | 0.012184994 | 1.314196021 | 0.143242288 | 2.193458545 | 0.028274358 | MAPK1 | 0.00209448 | 0.03030328 |
| 0.273140583 | 0.10901976 | 2.505422699 | 0.012230513 | 1.314084969 | 0.143261229 | 2.192393381 | 0.028351114 | RXRA | 0.003237621 | 0.03030328 |
| -0.272757719 | 0.109167607 | -2.49852246 | 0.012471224 | 0.761277208 | 0.083106811 | -2.872481662 | 0.004072617 | SHH | 0.002630301 | 0.030751014 |
| 0.272231093 | 0.109019728 | 2.497081009 | 0.012522035 | 1.312890366 | 0.143130951 | 2.186042669 | 0.028812484 | LAMB3 | 0.00338697 | 0.030751014 |
| 0.272215743 | 0.109335851 | 2.489720811 | 0.012784348 | 1.312870214 | 0.143543782 | 2.179615233 | 0.029285995 | EREG | 0.017039301 | 0.031067798 |
| -0.271518308 | 0.10907505 | -2.489279711 | 0.012800222 | 0.762221329 | 0.083139329 | -2.860002282 | 0.00423638 | TP73 | 0.002508629 | 0.031067798 |
| 0.271541452 | 0.109107149 | 2.488759482 | 0.012818965 | 1.311985255 | 0.143146971 | 2.179475074 | 0.029296394 | JUNB | 0.008536848 | 0.031067798 |
| -0.269635841 | 0.109030317 | -2.473035478 | 0.013397084 | 0.763657536 | 0.083261823 | -2.838545388 | 0.004531967 | LCN2 | 0.004639441 | 0.032229197 |
| 0.269889653 | 0.109153224 | 2.472576113 | 0.013414315 | 1.309819909 | 0.142971065 | 2.16701126 | 0.030233996 | MMP14 | 0.006881973 | 0.032229197 |
| 0.269102211 | 0.109095887 | 2.466657722 | 0.013638065 | 1.308788907 | 0.142783486 | 2.162637398 | 0.03056908 | SLC2A1 | 0.001326124 | 0.032625544 |
| 0.268163597 | 0.108959421 | 2.461132723 | 0.013849912 | 1.307561036 | 0.142471094 | 2.158760965 | 0.030868716 | CHUK | 0.002380532 | 0.032971174 |
| 0.26806187 | 0.108977001 | 2.45980223 | 0.01390136 | 1.307428029 | 0.142479586 | 2.157698781 | 0.030951259 | CREBBP | 0.002212429 | 0.032971174 |
| 0.266831693 | 0.109026418 | 2.447404021 | 0.014388944 | 1.305820649 | 0.142368948 | 2.148085341 | 0.031706977 | MAPK3 | 0.001240527 | 0.0339824 |
| 0.268905065 | 0.109945281 | 2.445808155 | 0.014452788 | 1.30853091 | 0.143866799 | 2.144559499 | 0.031988083 | ALX1 | 0.002401712 | 0.033988548 |
| 0.264307042 | 0.108959842 | 2.425728935 | 0.015277677 | 1.302528065 | 0.141923252 | 2.13163143 | 0.033037158 | CTNND1 | 0.002047246 | 0.035776837 |
| -0.262491834 | 0.109003335 | -2.408108291 | 0.016035424 | 0.769132645 | 0.083838023 | -2.7537309 | 0.005892019 | CXCL5 | 0.003739616 | 0.03739353 |
| 0.261565399 | 0.109141422 | 2.396573122 | 0.016549191 | 1.29896189 | 0.141770548 | 2.108772899 | 0.034964189 | NRP2 | 0.02187944 | 0.03843013 |
| 0.260972489 | 0.108988225 | 2.394501688 | 0.016642967 | 1.29819195 | 0.141487636 | 2.107547753 | 0.035070127 | RAB5A | 0.004535249 | 0.038486861 |
| 0.258750805 | 0.108991769 | 2.374039872 | 0.01759465 | 1.29531098 | 0.141178235 | 2.091759964 | 0.036459993 | KAT2B | 0.004639441 | 0.040353253 |
| 0.258527229 | 0.108898385 | 2.374022618 | 0.017595473 | 1.295021412 | 0.14102574 | 2.091968538 | 0.03644133 | DNMT1 | 0.001450508 | 0.040353253 |
| 0.258475751 | 0.108997123 | 2.371399756 | 0.017720853 | 1.294954748 | 0.141146342 | 2.089708766 | 0.036643969 | RGCC | 0.007907033 | 0.040473553 |
| 0.256899446 | 0.108966556 | 2.357599013 | 0.018393549 | 1.292915112 | 0.140884507 | 2.079115138 | 0.037606769 | UBC | 0.007534375 | 0.041837787 |
| 0.254413095 | 0.109043019 | 2.333144262 | 0.019640575 | 1.289704464 | 0.140633269 | 2.059999507 | 0.039398588 | TCF7L2 | 0.009100215 | 0.044491915 |
| 0.253157711 | 0.109006229 | 2.322415093 | 0.020210595 | 1.288086406 | 0.140409441 | 2.051759505 | 0.040193041 | MRTFA | 0.002976994 | 0.045597075 |
| 0.253130672 | 0.109097338 | 2.320227748 | 0.02032856 | 1.288051578 | 0.140522999 | 2.04985362 | 0.040378717 | U2AF1 | 0.003043673 | 0.045677534 |
| 0.253687697 | 0.10954147 | 2.315905531 | 0.020563427 | 1.288769255 | 0.141173679 | 2.045489335 | 0.04080664 | LIN28A | 0.024811672 | 0.04601896 |
| 0.250014365 | 0.109009302 | 2.293514049 | 0.021818427 | 1.284043862 | 0.139972725 | 2.029280078 | 0.042429771 | TGFB3 | 0.014403535 | 0.048631435 |
| 0.248203019 | 0.108927459 | 2.278608365 | 0.022690356 | 1.28172012 | 0.139614516 | 2.017842615 | 0.043607655 | SIRT1 | 0.004622827 | 0.050372591 |
| 0.248444356 | 0.109570391 | 2.267440625 | 0.023363322 | 1.282029483 | 0.140472472 | 2.007720655 | 0.044672984 | AGTR1 | 0.011062739 | 0.051659935 |
| 0.246118692 | 0.109029921 | 2.257350005 | 0.023986213 | 1.279051378 | 0.13945487 | 2.001015649 | 0.045390704 | JAK2 | 0.018624241 | 0.052826779 |
| -0.243976376 | 0.109086762 | -2.236535135 | 0.025316738 | 0.783506143 | 0.085470148 | -2.532976261 | 0.011309861 | TMPRSS2 | 0.007073793 | 0.055390442 |
| 0.243799326 | 0.109032265 | 2.236029179 | 0.02534986 | 1.276088227 | 0.13913479 | 1.98432202 | 0.047219953 | THBS1 | 0.031177827 | 0.055390442 |
| 0.240736703 | 0.109014018 | 2.208309611 | 0.027222698 | 1.272186029 | 0.138686111 | 1.96260482 | 0.04969211 | EGR1 | 0.017587276 | 0.059249401 |
| 0.240489283 | 0.109014488 | 2.206030466 | 0.027381867 | 1.271871303 | 0.138652399 | 1.960812102 | 0.049900946 | FGF2 | 0.020535325 | 0.059363031 |
| 0.237409412 | 0.109041613 | 2.177236796 | 0.029462904 | 1.26796013 | 0.138260418 | 1.938082743 | 0.052613128 | FZD4 | 0.009890611 | 0.063626116 |
| 0.232568912 | 0.108908697 | 2.135448476 | 0.032724395 | 1.261837399 | 0.137425067 | 1.905310318 | 0.056739745 | SIX1 | 0.005336774 | 0.0703955 |
| 0.232594842 | 0.109154489 | 2.130877477 | 0.033099237 | 1.261870119 | 0.137738788 | 1.901208243 | 0.057274742 | HGF | 0.031181102 | 0.070926937 |
| 0.231875865 | 0.10901841 | 2.126942262 | 0.033424881 | 1.260963189 | 0.137468203 | 1.898353104 | 0.057649583 | TIMP3 | 0.013297236 | 0.071174313 |
| 0.231587317 | 0.10891125 | 2.126385629 | 0.033471163 | 1.260599393 | 0.137293456 | 1.898119558 | 0.057680334 | BRAF | 0.005079071 | 0.071174313 |
| 0.230946552 | 0.109001456 | 2.118747406 | 0.03411182 | 1.259791904 | 0.137319152 | 1.891883987 | 0.05850644 | SPARC | 0.023475149 | 0.072259771 |
| 0.229673426 | 0.109073432 | 2.105677085 | 0.035232412 | 1.258189051 | 0.137234998 | 1.881364486 | 0.059922354 | SEMA3C | 0.048547088 | 0.074238913 |
| 0.229207808 | 0.108900591 | 2.104743462 | 0.035313645 | 1.257603352 | 0.136953749 | 1.880951445 | 0.059978524 | TMPRSS4 | 0.007173053 | 0.074238913 |
| 0.229228027 | 0.109020221 | 2.102619351 | 0.035499057 | 1.257628779 | 0.137106968 | 1.879034914 | 0.060239726 | CUX1 | 0.026704951 | 0.074325157 |
| 0.228938691 | 0.108955628 | 2.101210321 | 0.035622508 | 1.257264955 | 0.136986093 | 1.878037024 | 0.0603761 | MTR | 0.012819905 | 0.074325157 |
| 0.227062315 | 0.108899184 | 2.085069018 | 0.037063036 | 1.254908065 | 0.136658465 | 1.865292911 | 0.062140358 | ESRP2 | 0.012742317 | 0.076984962 |
| -0.227156185 | 0.109008456 | -2.083840032 | 0.037174721 | 0.796796325 | 0.086857537 | -2.339505371 | 0.019309294 | PROM1 | 0.022425088 | 0.076984962 |
| -0.224735768 | 0.109082109 | -2.060244061 | 0.039375215 | 0.79872724 | 0.087126852 | -2.310111691 | 0.020881971 | HEY2 | 0.007908108 | 0.081238826 |
| 0.222652073 | 0.108926236 | 2.044062846 | 0.040947339 | 1.249385803 | 0.136090893 | 1.832494421 | 0.066877792 | CLDN4 | 0.008435282 | 0.08416953 |
| 0.236310378 | 0.115988457 | 2.037361162 | 0.041613863 | 1.266567364 | 0.146907195 | 1.814528994 | 0.069596323 | KRT20 | 0.033852227 | 0.085223963 |
| 0.218925176 | 0.109067126 | 2.007251715 | 0.044722868 | 1.244738137 | 0.135760011 | 1.802726255 | 0.071431217 | PSMA3 | 0.007032332 | 0.091254381 |
| 0.218397089 | 0.108924898 | 2.005024496 | 0.044960428 | 1.244080981 | 0.135511394 | 1.801184189 | 0.071673854 | FGFR1 | 0.020765996 | 0.091403069 |
| 0.218233747 | 0.108931282 | 2.00340749 | 0.045133568 | 1.243877786 | 0.135497202 | 1.799873223 | 0.071880659 | DDX17 | 0.022257756 | 0.091420184 |
| 0.218013128 | 0.108929862 | 2.001408281 | 0.045348409 | 1.243603394 | 0.135465546 | 1.798268272 | 0.072134505 | ARRB1 | 0.008150259 | 0.091521335 |
| 0.217088183 | 0.108988121 | 1.991851793 | 0.046387322 | 1.242453661 | 0.135412689 | 1.790479623 | 0.07337684 | ITGB3 | 0.011624228 | 0.093278853 |
| 0.223856897 | 0.113946338 | 1.964581763 | 0.049462664 | 1.250892 | 0.142534562 | 1.760218688 | 0.078370735 | AFP | 0.047557989 | 0.099103895 |
| 0.213496187 | 0.108868961 | 1.961038162 | 0.049874571 | 1.237998777 | 0.13477964 | 1.76583627 | 0.077423322 | YWHAE | 0.008602187 | 0.099569738 |
